# HSPD1 repressed E-cadherin expression to promote cell invasion and migration for poor prognosis in oral squamous cell carcinoma

**DOI:** 10.1038/s41598-019-45489-1

**Published:** 2019-06-20

**Authors:** Bor-Hwang Kang, Chih-Wen Shu, Jian-Kang Chao, Cheng-Hsin Lee, Ting-Ying Fu, Huei-Han Liou, Luo-Ping Ger, Pei-Feng Liu

**Affiliations:** 1 Department of Otorhinolaryngology-Head and Neck Surgery, Kaohsiung Veterans General Hospital, Kaohsiung, Taiwan; 2 Graduate Institute of Aerospace and Undersea Medicine, National Defense Medical Center, Taipei, Taiwan; 3 Department of Pharmacy, Tajen University, Pingtung, Taiwan; 4 School of Medicine for International Students, I-Shou University, Kaohsiung, Taiwan; 5 Institute of Biomedical Sciences, National Sun Yat-sen University, Kaohsiung, Taiwan; 6 Department of Psychiatry, Pingtung Branch, Kaohsiung Veterans General Hospital, Pingtung, Taiwan; 7 Department of Medical Education and Research, Kaohsiung Veterans General Hospital, Kaohsiung, Taiwan; 8 Department of Pathology and Laboratory Medicine, Kaohsiung Veterans General Hospital, Kaohsiung, Taiwan; 9 Department of Oral Hygiene, Shu-Zen Junior College of Medicine and Management, Kaohsiung, Taiwan

**Keywords:** Tumour biomarkers, Oral cancer, Cell invasion

## Abstract

Buccal mucosa squamous cell carcinoma (BMSCC) is one of major subsites of oral cancer and is associated with a high rate of metastasis and poor prognosis. Heat shock proteins (HSPs) act as potential prognostic biomarkers in many cancer types. However, the role of HSPD1 in oral cancer, especially in BMSCC, is still unknown. Through data analysis with The Cancer Genome Atlas (TCGA), we found the association of HSPD1 gene expression with tumorigenesis and poor prognosis in oral cancer patients. Our cohort study showed that higher HSPD1 protein level was associated with tumorigenesis and poor prognosis in BMSCC patients with lymph node invasion, suggesting that HSPD1 may be involved in tumor metastasis. Moreover, knockdown of HSPD1 induced E-cadherin expression and decreased the migration and invasion of BMSCC cells. In contrast, ectopic expression of HSPD1 diminished E-cadherin expression and promoted the migration/invasion of BMSCC cells. Further, HSPD1 regulated RelA activation to repress E-cadherin expression, enhancing the migration and invasion of BMSCC cells. Furthermore, HSPD1 protein level was inversely correlated with E-cadherin protein level in tumor tissues and co-expression of high HSPD1/low E-cadherin showed a significant association with poor prognosis in BMSCC patients. Taken together, HSPD1 might repress E-cadherin expression and promote metastatic characters of BMSCC cells for poor prognosis of BMSCC patients.

## Introduction

Oral squamous cell carcinoma (OSCC) remains a major global health problem with increased incidence and poor 5-year overall survival^1,2^. Although OSCC is relatively easy to access for early diagnosis, it is an aggressive disease with the propensity for local recurrence and cervical lymph node metastasis^3^. OSCC accounts for 95% of all cancers in the oral cavity that includes the lip, tongue and buccal mucosa and the incidence of buccal mucosa squamous cell carcinoma (BMSCC) is higher in Southeast Asia use to betel quid chewing and tobacco smoking^4,5^. In North America and Western Europe, BMSCC also accounts for nearly 10% of cancer in oral cavity. BMSCC patients have a recurrence rate of up to 57% with associated low 5-year survival rates of approximately 50%. In addition, the incidence rate of cervical lymph node metastasis in BMSCC patients ranges from 25% to 54%^6^.

Heat shock proteins (HSPs) are groups of proteins involved in protein homeostasis under stresses and heat shock during normal physiology^7,8^. The major groups of HSPs classified by different molecular weight include HSPB1 (HSP27), DNAJB1 (HSP40), HSPD1(HSP60), HSPA4 (HSP70), HSP90AA1(HSP90) and HSPH (HSP110)^9^. Except normal cell protection, HSPs also play important roles in cancers development, progression, metastasis and drug resistance^10^. Potential clinical roles of several HSPs in oral cancers have been reported. For example: HSPA4 is considered as a prognostic indicator in OSCC^11^. HSP90AA1 and HSPB1 are prognostic biomarker and therapeutic target in OSCC^12,13^. HSP90B1 has potential clinical application as a novel diagnostic and prognostic biomarker for human OSCC^14^. HSPA5 is a potential biomarker for detection and treatment of oral cancer patients^9,15,16^. However, the clinical significance and molecular mechanism of HSPD1 in oral cancer is still not clear, particular in BMSCC.

Epithelial-to-mesenchymal transition (EMT), a process by the conversion of epithelial cells to a mesenchymal phenotype, is a key process linked to tumor metastasis^17–19^. The downregulation of E-cadherin required for polarity and cell-cell contacts is a hallmark of EMT^20^, which is related to poor prognosis in various cancer types^21^. The E-cadherin protein is downregulated in oral cancer cells compared with normal cells^22^. Importantly, low E-cadherin expression can predict lymph node metastasis in human OSCC cases and is considered an independent marker for survival in OSCC patients^23^. Moreover, E-cadherin can be transcriptionally repressed by several transcription factors, such as RelA and β-catenin^24,25^. The classical nuclear factor-kappa B (NF-κB), as a heterodimer of p50/p65 (RelA), translocate into the nucleus for E-cadherin repression^24^. Besides, β-catenin/T cell factor/lymphoid enhancer factor (TCF/LEF) transcription complex binds to target genes encoding repressors to downregulate E-cadherin expression^25^. These studies imply that the transcription factors may be involved in the regulation of E-cadherin for metastasis of OSCC.

In the present study, we indicated that HSPD1 regulated E-cadherin repression likely through RelA activation to promote cell migration and invasion of BMSCC cells. High HSPD1 was associated with poor prognosis in patients with lymph node invasion. In addition, according to The Cancer Genome Atlas (TCGA) database and our cohort, patients with high HSPD1 and low E-cadherin co-expression levels had shorter survival, suggesting that HSPD1 and E-cadherin conferred to metastasis and poor prognosis in BMSCC patients.

## Results

### The association of HSPD1 with tumorigenesis and survival in oral cancer patients according to TCGA dataset

To examine the clinical significance of HSPD1 in oral cancer, we analyzed gene expression levels of HSPD1 and several reported HSPs between 30 normal tissues and 315 tumor tissues in oral cancer patients with TCGA dataset. As data shown in Table 1, we found that expression levels of HSPD1 (p = 0.001), HSP90AA1 (p < 0.001), HSPE1 (p = 0.021), HSPH1 (p < 0.001), PSMA7 (p < 0.001), HSP90B1 (p < 0.001) and HSPA5 (p < 0.001) are significantly higher in tumor tissues. However, expression levels of DNAJB1 (p = 0.018), HSPB2 (p < 0.001), HSPB6 (p < 0.001) and HSPA1A (p = 0.021) were significantly lower in tumor tissues. Moreover, high expression level of HSPD1 (AHR = 1.76, 95% confidence interval (CI) = 1.06–2.92, p = 0.029, Table 1), HSP90AA1 (AHR = 1.47, 95% CI = 1.04–2.10, p = 0.032, Table 1) and HSAP5 (AHR = 1.77, 95% CI = 1.08–2.89, p = 0.023, Table 1) were significantly associated with poor OS but not with poor DFS. Although high expression level of HSPA4 (AHR = 1.65, 95% CI = 1.15–2.36, p = 0.006, Table 1) was also associated with poor OS, it was not associated with tumorigenesis in patients with oral cancer (p = 0.747, Table 1). The clinical oncogenesis roles of HSP90AA1 and HSPA5 in oral cancer have been reported as mentioned above. However, the clinical role of HSPD1 and its molecular mechanisms in cancer metastasis is not clear in oral cancer.

**Table 1:** The correlation of gene expression level of HSPs with tumorigenesis and survival of oral cancer patients from TCGA database.

ENTREZ gene symbol	Expression level of HSPs in tumor tissues compared to adjacent normal tissues		The association of high expression level of HSPs in tumor tissues with OS		The association of high expression level of HSPs in tumor tissues with DFS	
	Fold change (T/N)	p value*	AHR	*p value*^†^	AHR	*p value*^†^
HSPD1	**1.03**	**0.001**	**1.76 (1.06–2.92)**	**0.029**	1.30 (0.66–2.58)	0.448
HSP90AA1	**1.03**	**<0.001**	**1.47 (1.04–2.10)**	**0.032**	0.94 (0.56–1.58)	0.820
HSPA4	1.00	0.747	**1.65 (1.15–2.36)**	**0.006**	1.40 (0.83–2.36)	0.209
HSPB1	1.00	0.741	0.89 (0.57–1.37)	0.580	1.32 (0.65–2.69)	0.443
HSPE1	**1.03**	**0.021**	1.47 (1.00–2.18)	0.052	1.36 (0.79–2.34)	0.266
HSPH1	**1.07**	**<0.001**	1.38 (0.91–2.09)	0.134	1.00 (0.56–1.78)	0.992
PSMA7	**1.04**	**<0.001**	1.24 (0.87–1.76)	0.242	1.30 (0.77–2.20)	0.331
HSP90B1	**1.05**	**<0.001**	1.32 (0.89–1.93)	0.164	1.06 (0.61–1.84)	0.846
HSPA5	**1.04**	**<0.001**	**1.77 (1.08–2.89)**	**0.023**	0.93 (0.51–1.70)	0.808
DNAJB1	**0.98**	**0.018**	1.25 (0.88–1.77)	0.210	1.30 (0.77–2.18)	0.326
HSPB2	**0.77**	**<0.001**	1.22 (0.86–1.73)	0.266	1.57 (0.93–2.63)	0.090
HSPB6	**0.62**	**<0.001**	1.23 (0.87–1.75)	0.246	0.95 (0.56–1.60)	0.838
HSPA1A	**0.97**	**0.021**	1.10 (0.72–1.67)	0.663	0.66 (0.38–1.16)	0.150

Abbreviations: HSPs, heat shock proteins DFS, disease-free survival; OS, overall survival; AHR, adjusted hazard ratio.

N, normal tissues (n = 30); T, tumor tissues (n = 315).

*p values were estimated by Student’s t-test.

^†^p values were adjusted for cell differentiation (moderate + poor vs. well) and AJCC pathological stage (stage III + IV vs stage I + II) by multivariate Cox’s regression.

### The association of HSPD1 protein level with tumorigenesis and prognosis in BMSCC patients

Because BMSCC is the most common oral cavity cancer, we further investigated the clinical role of HSPD1 in BMSCC patients. HSPD1 protein level was compared between 128 corresponding tumor adjacent normal (CTAN) tissues and 186 BMSCC tissues on a tissue microarray (TMA) by immunohistochemistry (IHC). Similarly, the results showed that higher HSPD1 protein level was found in BMSCC tissues compared to the paired CTAN tissues (Supplementary Table S1, p < 0.001). Next, HSPD1 protein level and pathological outcome (T-classification, N-classification and cell differentiation) data were assessed using a Cox regression model for survival analysis. HSPD1 protein level showed an impact on disease-specific survival (DSS) [adjusted hazard ratio (AHR) = 3.85, 95% confidence interval (CI) = 2.28–6.50, p < 0.001, Table 2] and disease-free survival (DFS) (AHR = 2.00, 95% CI = 1.22–3.29, p = 0.006, Table 2) in BMSCC patients having N1 and N2 lymph node metastasis compared to those patients having no lymph node invasion (N0). These results revealed that HSPD1 protein level was associated with tumorigenesis and with poor prognosis in BMSCC patients with lymph node invasion.

**Table 2:** The correlation of protein level of HSPD1 and survival by different clinicopathological outcomes in BMSCC patients.

Variable		No. (%)	AHR (95% CI)	p value
**Disease-specific survival**				
T classification	T1, T2	141 (75.8)	1.00	
	T3, T4	45 (24.2)	1.42 (0.86–2.32)	0.168
N classification	N0	138 (74.2)	1.00	
	N1, N2	48 (25.8)	**3.85 (2.28–6.50)**	**<0.001**
Cell differentiation	Well	50 (26.9)	1.00	
	Moderate, poor	136 (73.1)	1.51 (0.80–2.87)	0.205
**Disease-free survival**				
T classification	T1, T2	141 (75.8)	1.00	
	T3, T4	45 (24.2)	0.79 (0.46–1.34)	0.379
N classification	N0	138 (74.2)	1.00	
	N1, N2	48 (25.8)	**2.00 (1.22–3.29)**	**0.006**
Cell differentiation	Well	50 (26.9)	1.00	
	Moderate, poor	136 (73.1)	1.60 (0.90–2.86)	0.110

Abbreviations: AHR, adjusted hazard ratio; BMSCC, buccal mucosa squamous cell carcinoma; CI, confidence interval.

p values were estimated by multivariate Cox’s regression.

p values were adjusted for cell differentiation (moderate + poor vs. well), T classification(T3 + T4 vs T1 + T2), and N classification(N1 + N2 vs N0).

### Involvement of HSPD1 in the migration and invasion of BMSCC cells

**Figure 1:**
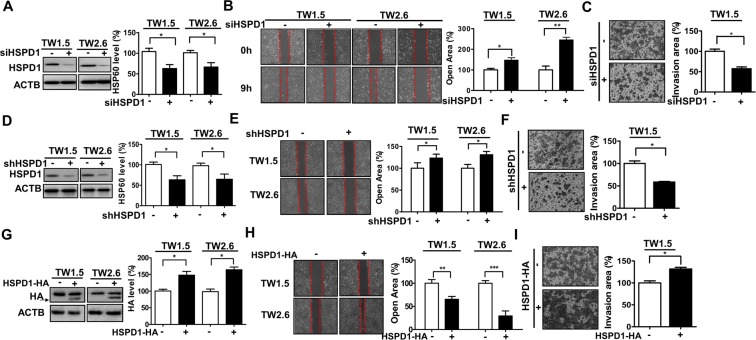
Effects of HSPD1 on the migration and invasion in TW1.5 and TW2.6 cells. The HSPD1 protein levels, migration and invasion of (**A–C**) HSPD1-silenced cells transfected with scrambled siRNA (5 nM, siCtrl) or siRNAs against HSPD1 (5 nM, siHSPD1), of **(D–F)** HSPD1 stable knockdown cells transfected with scrambled shRNA and shRNAs against HSPD1, and of (**G–I**) HSPD1-overexpressing cells transfected with the HA-tagged HSPD1 expression vector were analyzed. HA protein levels were analyzed by Western blot analysis. Abilities of migration and invasion were measured by wound-healing assay and transwell invasion assay, respectively. Full-length blots are presented in Supplementary information.

To verify the involvement of HSPD1 in metastatic characteristics, such as cell migration and invasion, in BMSCC, BMSCC cell lines TW1.5 and TW2.6 were transfected with siRNA and shRNA against HSPD1 or an expression vector encoding HA-tagged HSPD1. HSPD1 siRNA knockdown cells showed a 40% reduction in HSPD1 levels (Fig. 1A) and decreased migration (Fig. 1B) and invasion (Fig. 1C) abilities compared to cells transfected with scrambled siRNA. Similarly, stable HSPD1 shRNA knockdown cells showed a 40% reduction in HSPD1 levels (Fig. 1D) and had decreased migration and invasion abilities by 30% (Fig. 1E) and by 50% (Fig. 1F) compared to cells transfected by control shRNA, respectively. Conversely, HSPD1-overexpressing cells transfected with the HA-tagged HSPD1 expression vector showed a 50% increase in HSPD1 levels (arrow indicates HSPD1-fused HA-tag, Fig. 1G) and had higher migration and invasion abilities by 40–60% (Fig. 1H) and by 30% (Fig. 1I) compared to that of control cells, respectively. These results showed that HSPD1 may promote the migration and invasion of BMSCC cells.

### HSPD1 modulated E-cadherin repression in the migration and invasion of BMSCC cells

**Figure 2:**
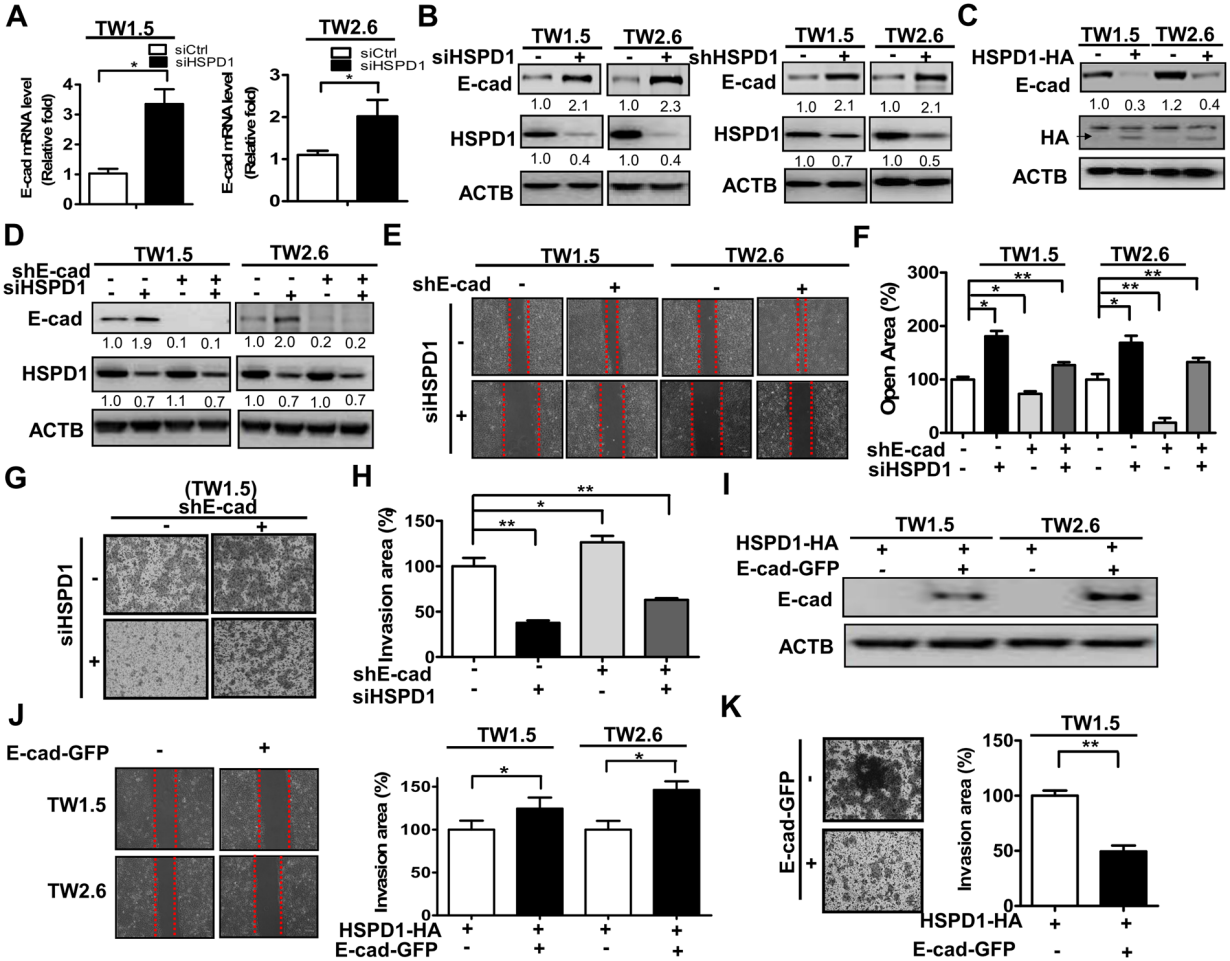
Effects of HSPD1 on E-cadherin repression and the migration and invasion of TW1.5 and TW2.6 cells. (**A**) E-cadherin gene expression was analyzed in cells transfected with scrambled siRNA or siRNA against HSPD1. (**B**) E-cadherin and HSPD1 protein levels were analyzed in HSPD1-silenced cells. **(C)** E-cadherin and HA protein levels were analyzed in cells transfected with the HA-tagged HSPD1 expression vector. **(D)** E-cadherin and HSPD1 protein levels were analyzed in E-cadherin stable knockdown cells harboring siRNAs against HSPD1. (**E-F**) The migration ability of E-cadherin stable knockdown cells harboring siRNAs against HSPD1 was analyzed. (**G-H**) The invasion ability of E-cadherin stable knockdown TW1.5 cells harboring siRNAs against HSPD1 was analyzed. (**I**) The HSPD1 protein level was analyzed in cells co-expressing GFP-tagged E-cadherin and HA-tagged HSPD1. (**J**) The migration ability of cells co-expressing GFP-tagged E-cadherin and HA-tagged HSPD1 was analyzed. (**K**) The cell invasion ability of TW1.5 cells co-expressing GFP-tagged E-cadherin and HA-tagged HSPD1 was analyzed. Gene expressions and protein levels were analyzed by RT-PCR and Western blot analysis, respectively. Abilities of migration and invasion were measured by wound-healing assay and transwell invasion assay, respectively. Full-length blots are presented in Supplementary information.

Low E-cadherin expression is associated with the invasiveness and metastatic potential of oral cancer cells. However, the role of HSPD1 in E-cadherin regulation of BMSCC cells is not understood. To verify the association of HSPD1 and E-cadherin with cell migration and invasion in BMSCC, HSPD1 was knockdowned by siRNA and shRNA in TW1.5 and TW2.6 cells and E-cadherin expression was analyzed by Real-Time PCR (RT-PCR) and Western blot analysis. The gene (Fig. 2A) and protein (Fig. 2B) expressions of E-cadherin were higher in HSPD1-knockdown cells, whereas E-cadherin expression was lower in HSPD1-overexpressing cells compared to that of control cells (Fig. 2C). Moreover, to investigate if HSPD1-regulated E-cadherin repression was involved in cell migration and invasion, E-cadherin stable knockdown TW1.5 and TW2.6 cells were established using shRNA (Fig. 2D). Reduced effects of silencing HSPD1 on migration and invasion were significantly recovered in E-cadherin stable knockdown cells in comparison to that control cells (Fig. 2E–H). Indeed, the cell migration of stable E-cadherin knockdowned cells was slightly reversed by siHSPD1, indicating that E-cadherin is not the only molecule modulated by HSPD1 for cell migration. Further, HSPD1-overexpressing cells transfected with the GFP-tagged E-cadherin expression vector (Fig. 2I) showed decreased migration and invasion by 25–40% (Fig. 2J) and by 50% (Fig. 2K) compared to control cells, respectively. These results indicated that HSPD1-regulated E-cadherin repression is involved in the migration and invasion of BMSCC cells.

### Involvement of RelA in HSPD1-modulated E-cadherin repression and migration/invasion of BMSCC cells

**Figure 3:**
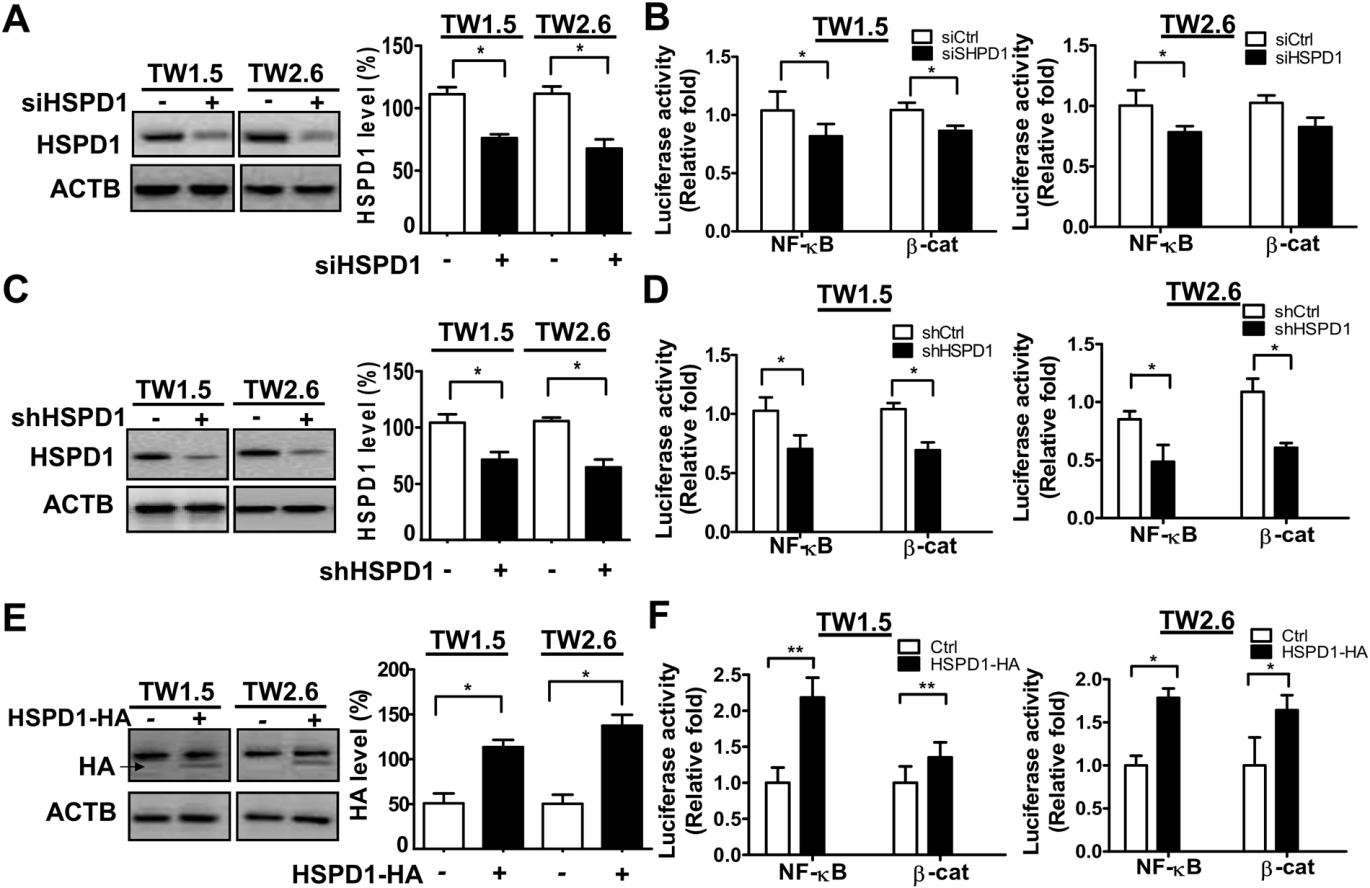
Effects of HSPD1 on NF-κB and β-catenin transcriptional activity in TW1.5 and TW2.6 cells. The transcriptional activities of NF-κB and β-catenin in (**A-B**) HSPD1-silenced cells with scrambled siRNA or siRNAs against HSPD1, in (**C-D**) HSPD1 stable knockdown cells with scrambled shRNA or shRNAs against HSPD1 infected cells, and in (**E-F**) HSPD1-overexpressing cells with the HA-tagged HSPD1 expression vector were analyzed. HSPD1 and HA protein levels were analyzed by Western blot analysis. The transcriptional activity was measured by luciferase assay. Full-length blots are presented in Supplementary information.

RelA (in the NF-κB pathway) and β-catenin (in the Wnt/β-catenin pathway) are repressors of E-cadherin expression and are required for the migration and invasion of cancer cells. To investigate whether the NF-κB pathway or Wnt/β-catenin pathway is activated by HSPD1, NF-κB- and β-catenin-mediated transcriptional activities were examined in HSPD1- knockdown and -overexpressing cells with luciferase assays. Both NF-κB- and β-cat-mediated transcriptional activities were reduced in HSPD1-knockdown cells (Fig. 3A,B; Fig. 3C,D) and increased in HSPD1-overexpressing cells (Fig. 3E,F), indicating that the NF-κB pathway and Wnt/β-catenin pathway were both activated by HSPD1.

**Figure 4:**
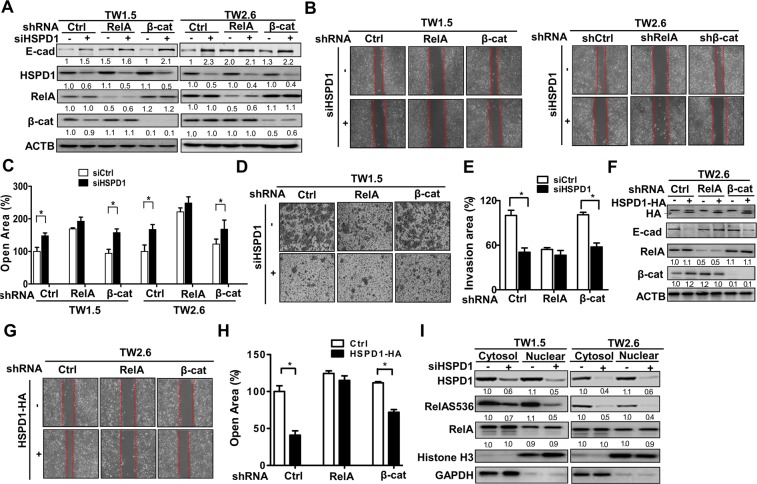
Involvement of HSPD1 in RelA-mediated migration and invasion through phosphorylation of RelA at Ser536 in TW1.5 and TW2.6 cells. **(A**) The protein levels of RelA and β-catenin stable knockdown TW1.5 and TW2.6 cells harboring siRNAs against HSPD1 were analyzed. (**B-C)** The migration abilities of RelA and β-catenin stable knockdown TW1.5 and TW2.6 cells harboring siRNAs against HSPD1 were analyzed. (**D-E)** The invasion abilities of RelA and β-catenin stable knockdown TW1.5 cells harboring siRNAs against HSPD1 were analyzed. (**F**) The protein levels of RelA and β-catenin stable knockdown TW2.6 cells harboring an HA-tagged HSPD1 expression vector were analyzed. (**G-H**) The migration abilities of RelA and β-catenin stable knockdown TW2.6 cells harboring an HA-tagged HSPD1 expression vector were analyzed and quantified. (**I**) RelAS536 phosphorylation and RelA expression in both the cytoplasm and nucleus of TW1.5 and TW2.6 cells transfected with HSPD1 siRNA were analyzed. HSPD1 protein levels were analyzed by Western blot analysis. The transcriptional activity was measured by luciferase assay. Full-length blots are presented in Supplementary information.

To further investigate if RelA or β-catenin is involved in HSPD1-regulated migration and invasion, RelA and β-catenin stable knockdown cells were established (Fig. 4A). Unlike control cells and stable β-catenin knockdowned cells, E-cadherin expression was elevated in RelA stable knockdowned cells with both scramble siRNA and siRNA against HSPD1 (Fig. 4A). Moreover, stable knockdown of RelA had no additive effects on silenced HSPD1-reduced migration and invasion in BMSCC cells (Fig. 4B–E). On the other hand, ectopic HSPD1 expression-reduced E-cadherin was recovered in RelA knockdowned cells, whereas it had no effects in β-cat stable knockdowned cells (Fig. 4F). Moreover, HSPD1 overexpression-promoted migration was reduced in RelA stable knockdown cells (Fig. 4F–H). These results indicated that HSPD1-regulated E-cadherin expression was mediated by RelA, which was involved in HSPD1-regulated migration and invasion.

To further evaluate if RelA is activated by HSPD1, protein levels of phosphorylated RelA from the cytoplasm and nucleus were compared in HSPD1-knockdown TW1.5 and TW2.6 cells. Our results indicated that the level of phosphorylated RelAS536 was decreased in both the cytoplasm and nucleus of HSPD1-knockdown cells (Fig. 4I). Taken together, HSPD1-regulated E-cadherin repression in the migration and invasion of BMSCC cells, which might be through phosphorylation and transcriptional activity of RelA.

### The association of HSPD1/E-cadherin co-expression with prognosis in BMSCC patients

**Figure 5:**
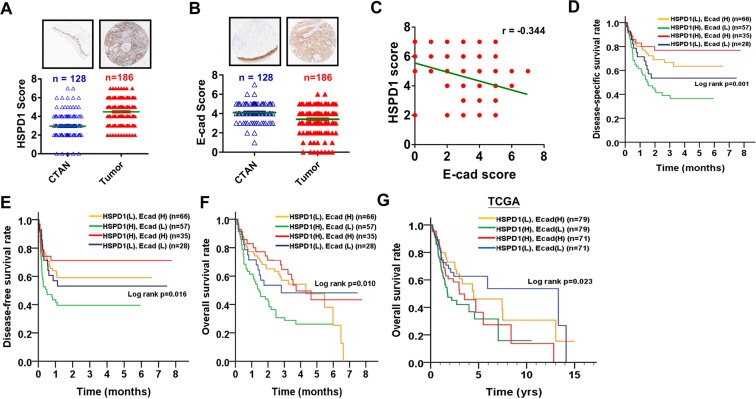
Correlation analysis of HSPD1 and E-cadherin protein levels in tumor tissues and survival curves according to co-expression level of HSPD1 and E-cadherin in oral cancer patients. (**A**) The HSPD1 protein levels determined by IHC were compared between CTAN and tumor tissues in BMSCC patients. (**B**) The E-cadherin protein levels determined by IHC were compared between CTAN and tumor tissues in BMSCC patients. (**C**) The correlation of HSPD1 and E-cadherin protein levels in BMSCC patients was analyzed. (**D-E**) The Kaplan-Meier curves were analyzed for DSS and DFS in BMSCC patients with co-expression level of HSPD1 and E-cadherin. **(F)** The Kaplan-Meier curves were analyzed for OS in BMSCC patients with co-expression level of HSPD1 and E-cadherin. (**G**) The Kaplan-Meier curves were analyzed for OS of TCGA oral cancer patients with co-expression level of HSPD1 and E-cadherin. (H, means high protein level of HSPD1 or E-cadherin; L, means low protein level of HSPD1 or E-cadherin).

Our results demonstrated that HSPD1-regulated E-cadherin repression was associated with migration and invasion of BMSCC cells (Fig. 2). To examine the correlation of HSPD1 and E-cadherin in BMSCC patients, we evaluated the protein levels of HSPD1 and E-cadherin with TMA and found that protein level of HSPD1 was higher (Fig. 5A, p < 0.001; Supplementary Table S1, p < 0.001) and protein level of E-cadherin was lower (Fig. 5B, p < 0.001; Supplementary Table S1, p < 0.001) in BMSCC tissues in comparison to the CTAN tissues. Moreover, protein level of HSPD1 negatively correlated with protein level of E-cadherin in BMSCC tissues (Fig. 5C; correlation coefficient (r) = −0.344, p < 0.001). To determine whether the co-expression levels of HSPD1 and E-cadherin were involved in the survival of BMSCC patients, the Kaplan-Meier method and Cox proportional hazards models were used. The co-expression level of high HSPD1/low E-cadherin was significantly associated with poor DSS (log rank p = 0.001, Fig. 5D; AHR = 2.24, 95% CI = 1.32–3.83, p = 0.003, Table 3) and DFS (log rank p = 0.016, Fig. 5E; AHR = 1.88, 95% CI = 1.13–3.14, p = 0.015, Table 3) in BMSCC patients. Moreover, BMSCC patients with co-expression level of high HSPD1/low E-cadherin had poor overall survival (OS) (log rank p = 0.010, Fig. 5F; AHR = 1.88, 95% CI = 1.18–2.98, p = 0.008, Supplementary Table S2). Similarly, TCGA data analysis showed that oral cancer patients with co-expression level of high HSPD1/low E-cadherin also had poor OS (log rank p = 0.023, Fig. 5G; AHR = 1.86, 95% CI = 1.15–3.01, p = 0.012, Supplementary Table S3). These data indicated that co-expression level of high HSPD1/low E-cadherin was associated with poor prognosis of BMSCC patients.

**Table 3:** The association of co-expression level of HSPD1 and E-cad with survival in BMSCC patients.

Variable	No. (%)	CHR (95% CI)	p value*	AHR (95% CI)	p value^†^
**Disease-specific survival**					
HSPD1 (L) E-cad (H)	66 (35.5)	1.00		1.00	
HSPD1 (H) E-cad (L)	57 (30.6)	**2.24 (1.43–3.51)**	**<0.001**	**2.24 (1.32–3.83)**	**0.003**
HSPD1 (H) E-cad (H)	35 (18.8)	0.43 (0.21–0.90)	0.025	0.65 (0.29–1.46)	0.300
HSPD1 (L) E-cad (L)	28 (15.1)	1.14 (0.63–2.08)	0.658	1.49 (0.75–2.96)	0.253
**Disease-free survival**					
HSPD1 (L) E-cad (H)	66 (35.5)	1.00		1.00	
HSPD1 (H) E-cad (L)	57 (30.6)	**1.96 (1.26–3.04)**	**0.003**	**1.88 (1.13–3.14)**	**0.015**
HSPD1 (H) E-cad (H)	35 (18.8)	0.55 (0.29–1.07)	0.078	0.73 (0.35–1.51)	0.392
HSPD1 (L) E-cad (L)	28 (15.1)	0.96 (0.53–1.73)	0.888	1.16 (0.60–2.26)	0.662

Abbreviations: AHR, adjusted hazard ratio; BMSCC, buccal mucosa squamous cell carcinoma; CHR, crude hazard ratio; CI, confidence interval; E-cad, E-cadherin; H, High expression; L, Low expression.

*p values were estimated by Cox’s regression.

^†^p values were estimated by multivariate Cox’s regression.

^†^p values were adjusted for cell differentiation (moderate + poor vs. well), T classification (T3 + T4 vs T1 + T2), and N classification (N1 + N2 vs N0).

## Discussion

HSPD1, a nuclear-encoded mitochondrial protein association with co-chaperonin HSP10, helps fold proteins to facilitate degradation of misfolded or denatured proteins, which involved in the activation of the immune system, has pro-inflammatory functions, and has pro-survival or pro-apoptotic roles^26^. Moreover, abnormalities in expression and subcellular localization of HSPD1 were also related to neurodegenerative disorders, inflammatory diseases and various cancers. Most studies showed that overexpression of HSPD1 is associated with cancer progression in various tumors^16,27–31^. However, its clinical role and molecular mechanisms in oral cancer remains unclear, especially in BMSCC. In the study, we reported the following findings: First, we found HSPD1 gene expression was associated with both tumorigenesis and poor prognosis in oral cancer patients from TCGA database. Second, HSPD1 protein level was associated with tumorigenesis and poor survival in BMSCC patients. Third, HSPD1 was involved in the migration and invasion of BMSCC cells, likely through RelA activation and E-cadherin repression. Fourth, co-expression level of high HSPD1 and low E-cadherin was highly correlated with poor prognosis in BMSCC patients, suggesting these molecules could be potential prognostic factors for BMSCC.

HSPD1 is a molecular chaperone localized mainly in the mitochondrial matrix^32^, but it is recently found in many extramitochondrial sites, such as the outer mitochondrial surface, the cell surface, the intracellular vesicles, nucleus, the extracellular space, and even in the cytosol. Recently, exosomal HSPD1 is reported as a potential diagnostic and prognostic biomarker in cancer, especially in colorectal cancer^33^. Accumulating evidence has demonstrated that HSPD1, especially cytosolic HSPD1, is involved in survival and the metastasis of various cancers^27,28^. One group indicated that cytosolic HSPD1 interacts with β-catenin to promote metastasis by increasing the protein levels and transcriptional activity of β-catenin in head and neck cancer^31^, which may led to the accumulation of free β-catenin to activate mesenchymal gene expression^34^. Moreover, cytosolic HSPD1 activates NF-κB by directly interacting with IκB kinase (IKK) α/β for cell survival^35^. However, its molecular mechanism in metastatic cancer is still unclear. Our studies first indicated that HSPD1 might promote cell invasion and migration by increasing the transcriptional activation of RelA to repress E-cadherin in BMSCC, while the localization of cytosolic HSPD1 in RelA regulation will need further investigation. Astonishingly, there is an opposite finding that HSPD1 acts as a tumor suppressor to inhibit invasion by increasing E-cadherin in hepatocellular carcinoma^30^, likely due to different mechanisms in various cancer types.

Our results showed that HSPD1 repressed E-cadherin expression at transcriptional and translational levels in BMSCC (Fig. 2). In addition to the repression of E-cadherin by HSPD1 through RelA activation, E-cadherin could be repressed by several factors, including SNAIL, ZEB1, ZEB2, SLUG and TWIST^36–38^. However, the involvement of SNAIL, SLUG and TWIST in HSPD1-mediated E-cadherin repression was not observed in BMSCC cells (Supplementary Fig. S1), implying that HSPD1-mediated EMT process may be regulated by other pathways^16,39^. On the other hand, E-cadherin could also repress the nuclear localization of NF-κB, affecting its transcriptional activity^40^, and a loss of E-cadherin leads to the induction of NF-κB activity in the cell^41^. Thus, the reciprocal repression between E-cadherin and RelA on HSPD1-modulated metastasis needs to be verified in the future.

In the study, we would like to investigate the role of HSPD1 in cell migration/invasion. Thus, we measured several important EMT-markers such as E-cadherin, N-cadherin, Snail, Twist, Slug and c-Myc in HSPD1-knockdowend cells and found that only E-cadherin was significantly regulated by HSPD1 (Supplementary Fig. S1). Moreover, previous studied indicated that the downregulation of E-cadherin required for polarity and cell-cell contacts is a hallmark of EMT and low E-cadherin expression has been considered an independent marker for survival in OSCC patients. Thus, we focused on estimating the change of HSPD1-regulated E-cadherin expression and found that that E-cadherin was one of potential EMT-markers for HSPD1-mediated metastasis in OSCC. Definitely, other EMT-markers involving in HSPD1-mediated metastasis could not be excluded.

Our results indicated that co-expression level of high HSPD1/low E-cadherin was significantly associated with poor survival in BMSCC patients (Fig. 5 and Table 3). Analysis with another independent cohort from TCGA database also showed that oral cancer patients with co-expression level of high HSPD1/low E-cadherin had poor OS compared to those with co-expression level of low HSPD1/high E-cadherin (Fig. 5G and Supplementary Table S3). Moreover, our results indicated that cells with low HSPD1/low E-cadherin showed lower migration ability compared to cells with high HSPD1/low E-cad (Fig. 2E,F). Furthermore, patients with low HSPD1/low E-cadherin expression still have better survival [DSS AHR = 1.49 (0.75–2.96); DFS AHR = 1.16 (0.60–2.26), Table 3] compared to patients with high HSPD1/low E-cadherin expression [DSS AHR = 2.24 (1.32–3.83); DFS AHR = 1.88 (1.13–3.14), Table 3], which supports the notion that low HSPD1/low E-cadherin expression has protective effects in oral cancer patients although it is not significant different. The possible reason might be i) cohort limitation ii) heterogenous and complicated microenvironment of tumors *in vivo*. On the other hand, patients with high HSPD1/high RelA also showed poor OS than those with co-expression level of low HSPD1/low RelA (AHR = 1.98, 95% CI = 1.11–3.55, p = 0.021, Supplementary Table S4). These analyzed data might imply the importance of high HSPD1/low E-cadherin/high RelA expressions in the prognosis of BMSCC patients.

Except epithelial E-cadherin, epithelial N-cadherin, nuclear Snail and Twist and cytoplasmic Vimentin were also well-known biomarker markers for EMT process in cancers^42^. To investigate if these EMT markers also involve in HSPD1-mediated the expression, the expression correlation between these EMT markers and HSPD1 was analyzed. We found that protein level of E-cadherin was the only EMT marker negatively correlated with protein level of HSPD1 (r = −0.327, p < 0.001, Supplementary Table S5), highlighting the HSPD1 repressed E-cadherin expression is specifically critical for metastasis and poor prognosis in BMSCC.

Notably, survival rate of oral cancer patients with co-expression level of low HSPD1/low E-cadherin is higher than those with co-expression level of high HSPD1/high E-cadherin from TGCA cohort (Fig. 5G), which is opposite to the findings in our cohort study (Fig. 5D–F). Our cohort study was based on protein level in BMSCC patients, whereas TCGA database analysis was based on mRNA level. Moreover, oral cancer cohort from TCGA database only includes 24 BMSCC patients and most of oral patients are TSCC patients. Thus, co-expression level of HSPD1 and E-cadherin plays different role between our cohort and TCGA might be due to (1) different molecular level (2) different subsites of tumor tissues.

HSPD1 is related to chronic inflammation, which may increase cancer metastasis^43^. Moreover, the buccal mucosa is the site with the highest risk of contracting a malignancy in patients exposed to common inflammation-related carcinogens, such as cigarettes, betel quid, and alcohol^44^. In our stratified analysis, we found a possible association of higher HSPD1 expression with BMSCC patients who chewed betel quid (p = 0.009, Supplementary Table S6) but not who smoked or drank alcohol (Supplementary Table S6). Moreover, HSPD1 expression is associated with recurrence in BMSCC patients, implying that HSPD1 may mediate drug resistance and could be a prognostic factor for cancer therapy^45^. Sphere-forming stem-like cell populations have the potential for chemoresistance or radioresistance in human sarcoma cell lines^46^. However, cell viability in 3D cell spheres was not significantly different between the control and HSPD1-knockdown cells (Supplementary Fig. S2). The possible roles of HSPD1 in chemoresistance or radioresistance in the recurrence of BMSCC still require further verification. The HSPD1 repressed E-cadherin expression through RelA activation may be involved in metastasis in BMSCC cells, and co-expression of high HSPD1/low E-cadherin could be a potential prognostic biomarker for BMSCC patients.

## Methods

### Clinical samples

The specimens of 128 CTAN and 186 BMSCC tissues from 1993 to 2006 were harvested in Kaohsiung Veterans General Hospital. These informed consents were provided by all patients and the study was approved by the Institutional Review Board at Kaohsiung Veterans General Hospital (IRB number: VGHKS11-CT12-13). All studies involving human participants were in accordance with the ethical standards of the institutional and/or national research committee.

### TMA block construction

The representative area of tumor and CTAN tissues was selected and the TMA blocks were constructed as described previously^47^. Basically, TMA block was composed of 48 trios. Each trio contained two cores from the tumor tissue and one core from the CTAN of the same patient. Five cores of normal uvula epithelium from other persons were also included in each TMA block. Cores with incorrect content were excluded. therefore, total 7 TMA blocks were constructed. TMA blocks were cut into 4-μm serial sections, deparaffinized in xylene, rehydrated in gradient ethanol, and washed for 5 min with phosphate-buffered saline for further IHC staining.

### IHC analysis and scoring

IHC staining for all tissues were performed by the Novolink Max Polymer Detection System (Leica, Newcastle Upon Tyne, United Kingdom). Antigen retrieval was accessed by a pressure boiler in Tris-EDTA (10 mM, pH 9.0) for 10 min, and endogenous peroxidase was blocked with 3% hydrogen peroxide at room temperature for 10 min. The slides were incubated with anti-HSPD1 (dilution 1:200; Abcam Inc. Cambridge, MA, USA) and anti-E-cadherin (dilution 1:200; BD Bioscience, San Diego, CA, USA) antibodies overnight at 4 °C in a moisture chamber. After washing with PBS, the slides were incubated with secondary antibody labeled with horseradish peroxidase at room temperature for 10 min and then counterstained with hematoxylin.

A semiquantitative approach was used to grade immunoreactivity. First, an oral cancer pathologist (Ting-Ying Fu) accompanied two pathology technicians (Cheng-Hsin Lee and Huei-Han Liou) evaluated the slides to resolve all discrepancies. Total 5–20% of the core samples were randomly selected by the oral cancer pathologist for re-evaluation after they independently reviewed all of the slides. The staining intensity of cytoplasmic HSPD1 and membrane E-cadherin staining was measured using a numerical scale indicated as 0 (negative expression), 1 (weak expression), 2 (moderate expression) and 3 (strong expression) (Supplementary Fig. S3). The percentage of cells staining was scored as 0 (<5%), 1 (5–25%), 2 (26–50%), 3, (51–75%), and 4 (>75%). The final scores (0–7) was the sum of the score for intensity (0–3) added to the score for percentage (0–4). Based on the distribution of HSPD1 and E-cadherin scores, the low and high expression levels were dichotomized by the cutoff set at the 50th percentile with cutoff values 4 and 3 for HSPD1 and E-cadherin, respectively. The low and high gene expression levels from TCGA were dichotomized based on a receiver operating characteristic curve analysis.

### Vectors

An NF-κB responsive promoter vector (pGL4.32), a TCF*/*LEF responsive promoter vector (pGL4.49) and a CMV constitutive promoter vector (pGL4.50) were purchased from Promega (Promega Corporation, Madison, WI, USA). The pCGN-HA expression vector encoding full-length cDNA of HSPD1 was a gift from Dr. Sang Won Kang at Ewha Womans University in Korea. A GFP-tagged E-cadherin expression vector was purchased from Addgene (plasmid # 28009).

### Cell culture

Buccal mucosa oral cancer-derived cell lines (TW 1.5 and TW 2.6)^48,49^, provided from Dr. Michael Hsiao at Academia Sinica in Taiwan and Dr. Mark Yen-Ping Kuo at National Taiwan University Hospital, were grown in Dulbecco’s modified Eagle’s medium (DMEM) (Invitrogen-Gibco, Carlsbad, CA, USA) with 10% heat-inactivated fetal bovine serum (Biological Industries, Kibbutz, Israel), with 100 U/ml penicillin (Invitrogen-Gibco, Carlsbad, CA, USA), and with 100 μg/ml streptomycin (Invitrogen-Gibco, Carlsbad, CA, USA) at 37 °C in a humidified 5% CO_2_ atmosphere. Cells were grown on Corning tissue culture plates (Corning Incorporated, Corning, CA, USA).

### Cell transient transfection

Oral cancer cells were seeded into 6-well plates then transfected with the pCGN-HA vector containing full-length cDNA of human HSPD1 or with the GFP-tagged E-cadherin expression vector using X-tremeGENE (Sigma-Aldrich Corporation, St. Louis, MO, USA) for 24 h. For transient knockdown, cells were transfected with 5 nM scrambled siRNA or siRNA against HSPD1, RelA, β-catenin, and E-cadherin (Ambion, Foster City, CA, USA) using RNAiMAX (Life technologies, Carlsbad, CA, USA) for 48 h.

### Cell stable selection

shRNAs against HSPD1 (TRCN0000029446), RelA (TRCN0000014684), β-catenin (TRCN0000003845) and E-cadherin (TRCN0000237843) were purchased from The RNAi Consortium (TRC, Taiwan). Total vectors (2 μg) were transfected into 1 × 10^6^ HEK293FT cells using 2 μl of Lipofectamine 2000 (Thermo Fisher Scientific, Waltham, MA, USA). The culture supernatant was harvested on days 2, and the cell debris was removed for further infection of TW2.5 and TW2.6 cells. The infected TW2.5 and TW2.6 cells were selected with 1–3 μg/ml puromycin for at least 10 days to obtain stable cell lines. The knockdown efficiency in cells stably harboring shRNAs against HSPD1, RelA and β-catenin was verified by immunoblotting. All experiments were performed as approved by the Biosafety Committee at Kaohsiung Veterans General Hospital.

### RT-PCR

Total RNA of TW2.5 and TW2.6 cells transfected with siRNA against HSPD1 was extracted with TRIzol reagent (Invitrogen, Carlsbad, CA, USA). Then, 1 μg of total RNA was reverse transcribed with SuperScript II RNase H-Reverse Transcriptase (Invitrogen, Carlsbad, CA, USA) for cDNA synthesis. The amount of target gene mRNA relative to actin was analyzed by RT-PCR performed in a StepOnePlusTM system (ABI Prism 7000 sequence detection system) with SYBR Green Master Mix (Applied Biosystems, Foster City, CA, USA). A comparative cycle threshold method was used for quantifying the fluorescence signals.

### Immunoblotting

After PBS rinsed, the cells were lysed in RIPA buffer (1% NP40, 50 mM Tris-HCl pH 7.5, 150 mM NaCl, 0.25% sodium deoxycholate, 0.1% SDS) plus protease inhibitor cocktail. The protein samples from the cells were separated by a 10% (w/v) SDS-PAGE gel and electrophoretically transferred from the gel to nitrocellulose membranes (Millipore, Billerica, MA, USA). The membranes were blocked in 5% (w/v) skim milk and then incubated overnight at 4 °C with primary antibodies against HSPD1 (Abcam, MA, USA), E-cadherin, RelA, RelAS536-P, β-catenin, H3 histone, GAPDH (Cell Signaling Technology Inc. Danvers, MA, USA), hemagglutinin (HA) tag (Roche Diagnostics, Rotkreuz, AG, Switzerland) and ACTB (β-actin) (Sigma-Aldrich Corporation, St. Louis, MO, USA). The proteins were probed with horseradish peroxidase-labeled secondary antibody and detected with an ECL reagent. The protein expression level on the membrane was analyzed and measured with the ChemiDoc XRS Imaging System (Bio-Rad) and ImageJ software.

### Luciferase reporter assay

To test RelA and β-catenin transcriptional activity, cells (8 × 103 cells/50 μl) were transfected with 2 μg of the NF-κB responsive promoter vector (pGL4.32) and TCF-LEF responsive reporter vector (pGL4.49) for 16 h in 96-well plates. The ONE-Glo® Luminescent Cell Viability Assay kit (Promega Corporation, Madison, WI, USA) and a Fluoroskan Ascent FL reader (Thermo Fisher Scientific, Waltham, USA) were used for measuring and quantifying the luminescence of the luciferase-based reporter, respectively. CMV constitutive luciferase expression (pGL4.50) was used to normalize luminescent signals^50^.

### Wound-healing assay

Culture dishes were fitted with IBIDI Culture-Inserts (35 mm with high culture-insert coating). Suspensions of cells in FBS-free DMEM (140 µl) were seeded at a density of 1.5 × 10^5^ cells/ml in the insert and cultured at 37 °C with 5% CO_2_ overnight. Subsequently, culture inserts were removed, and wound healing as an indication of cancer migration was observed for 9 h. Migration distance was measured in triplicate.

### Transwell invasion assay

The assay was performed using transwell inserts with 8 μm pore (Greiner Bio-One, St. Louis, MO, USA). A total of 8 × 10^4^ cells in 300 µl DMEM containing 1% FBS were seeded into the top chamber of 0.5% Matrigel-coated transwell inserts (Collaborative Research Inc., Bedford, MA, USA). To stimulate cell invasion, complete medium was added to the bottom of transwell inserts. After 24 h, the cells on the upper side of the filter were removed, and the cells adhering to the bottom surface of the filter were fixed in 4% formaldehyde and stained with 0.1% crystal violet. The cells invading from matrigel to the reverse side of filter were counted under a microscope (magnification of × 200) in five random fields. All invasion assays were performed in triplicate.

### Subcellular fractionation

The subcellular fractions were obtained after differential centrifugation. Briefly, cells were centrifuged, and the pelleted cell were resuspended in ice-cold fractionation buffer (10 mM HEPES pH = 7.9, 10 mM KCl, 0.1 mM EDTA, 0.1 mM EGTA, 1 mM dithiothreitol, protease inhibitors mixture, 1 mM NaF, 1 mM Na_3_VO_4_, and 50 mM β-glycerophosphate). After rupturing the cells, the cells and debris were removed by centrifugation at 500 g for 10 min and the supernatant was subsequently separated into pellets (nuclear fraction) and supernatants (cytosol fraction) by centrifugation at 10,000 g for 10 min.

### TCGA dataset and statistical analysis

All statistical analysis was performed with SPSS software (version 20.0, SPSS Inc., Chicago, USA). RNA-sequencing transcriptome profiles of all genes were downloaded from the public TCGA data portal (https://cancergenome.nih.gov). The gene expression data of 30 normal and 315 tumor tissues of oral cancer patients were used to analyze the association of HSPs expression with tumorigenesis. Also, the gene expression data of 315 tumors tissues of oral cancer patients from TCGA database were used for analyzing the impact of gene expression in survival. The hazard ratio was adjusted for cell differentiation (moderate + poor vs. well) and AJCC pathological stage (stage III + IV vs. stage I + II) by multivariate Cox’s regression. In our cohort, DSS was measured as the time from date of the primary surgery to the date of cancer-specific death or the last follow-up. DFS, included both local and regional DFS, was calculated as the time from the date of the primary surgery to the date of first recurrence or the last follow-up. Kaplan-Meier method, log-rank test, and Cox’s proportional hazards model were used for survival analysis. The hazard ratio was adjusted for cell differentiation (moderate + poor vs. well), T classification (T3 + T4 vs T1 + T2), and N classification (N1 + N2 vs N0). A P-value of 0.05 or less was considered significant (*), a P-value of 0.01 or less was considered highly significant (**), and a P-value of 0.001 or less was considered extremely significant (***). All quantified results are expressed as the mean ± SEM from 3 individual experiments.

## Supplementary information

Supplementary Information

## References

[1] Ishida K (2017). Current mouse models of oral squamous cell carcinoma: Genetic and chemically induced models. Oral Oncol.

[2] Manikandan M (2016). Oral squamous cell carcinoma: microRNA expression profiling and integrative analyses for elucidation of tumourigenesis mechanism. Mol Cancer.

[3] Pramanik KK (2018). Glycogen synthase kinase-3beta mediated regulation of matrix metalloproteinase-9 and its involvement in oral squamous cell carcinoma progression and invasion. Cell Oncol (Dordr).

[4] Saini, J. & Sharma, P. K. Clinical, Prognostic and Therapeutic Significance of Heat Shock Proteins in Cancer. *Curr Drug Targets* **19** (2017). 10.2174/1389450118666170823121248 28831912

[5] Fu TY (2011). Manganese superoxide dismutase and glutathione peroxidase as prognostic markers in patients with buccal mucosal squamous cell carcinomas. Head Neck.

[6] Bachar G (2012). Squamous cell carcinoma of the buccal mucosa: outcomes of treatment in the modern era. Laryngoscope.

[7] Wu J (2017). Heat Shock Proteins and Cancer. Trends Pharmacol Sci.

[8] Ren, D., Fan, M., Sun, C., Zhou, C. & Li, Y. Capillary Electrophoresis with Laser Induced Fluorescence Detection for Study of the Association of HSP60 Gene Polymorphism with Gouty Arthritis. *J AOAC Int* (2018). 10.5740/jaoacint.18-0242 30340651

[9] Sannam Khan, R., Khurshid, Z., Akhbar, S. & Faraz Moin, S. Advances of Salivary Proteomics in Oral Squamous Cell Carcinoma (OSCC) Detection: An Update. *Proteomes* **4** (2016). 10.3390/proteomes4040041 PMC5260973 28248250

[10] Calderwood SK, Gong J (2016). Heat Shock Proteins Promote Cancer: It’s a Protection Racket. Trends Biochem Sci.

[11] Taghavi N, Mohsenifar Z, Baghban AA, Arjomandkhah A (2018). CD20+ Tumor Infiltrating B Lymphocyte in Oral Squamous Cell Carcinoma: Correlation with Clinicopathologic Characteristics and Heat Shock Protein 70 Expression. Patholog Res Int.

[12] Ono, K. *et al*. HSP-enriched properties of extracellular vesicles involve survival of metastatic oral cancer cells. *J Cell Biochem* **(**In Press**)** (2018). 10.1002/jcb.27039 29768689

[13] Zheng G (2018). HSP27-Mediated Extracellular and Intracellular Signaling Pathways Synergistically Confer Chemoresistance in Squamous Cell Carcinoma of Tongue. Clin Cancer Res.

[14] Nomura H (2007). Network-based analysis of calcium-binding protein genes identifies Grp94 as a target in human oral carcinogenesis. Br J Cancer.

[15] Xia F (2014). Glucose-regulated protein 78 and heparanase expression in oral squamous cell carcinoma: correlations and prognostic significance. World J Surg Oncol.

[16] Yu JS (2016). Saliva protein biomarkers to detect oral squamous cell carcinoma in a high-risk population in Taiwan. Proc Natl Acad Sci USA.

[17] Zhou D (2016). RBP2 induces stem-like cancer cells by promoting EMT and is a prognostic marker for renal cell carcinoma. Exp Mol Med.

[18] Karaosmanoglu O, Banerjee S, Sivas H (2018). Identification of biomarkers associated with partial epithelial to mesenchymal transition in the secretome of slug over-expressing hepatocellular carcinoma cells. Cell Oncol (Dordr).

[19] Vu, T., Jin, L. & Datta, P. K. Effect of Cigarette Smoking on Epithelial to Mesenchymal Transition (EMT) in Lung Cancer. *J Clin Med* **5** (2016). 10.3390/jcm5040044 PMC4850467 27077888

[20] Chen IC (2014). Role of SIRT1 in regulation of epithelial-to-mesenchymal transition in oral squamous cell carcinoma metastasis. Mol Cancer.

[21] Serrano-Gomez SJ, Maziveyi M, Alahari SK (2016). Regulation of epithelial-mesenchymal transition through epigenetic and post-translational modifications. Mol Cancer.

[22] Chaw SY (2012). Epithelial to mesenchymal transition (EMT) biomarkers–E-cadherin, beta-catenin, APC and Vimentin–in oral squamous cell carcinogenesis and transformation. Oral Oncol.

[23] Andl T (2014). Concerted loss of TGFbeta-mediated proliferation control and E-cadherin disrupts epithelial homeostasis and causes oral squamous cell carcinoma. Carcinogenesis.

[24] Chua HL (2007). NF-kappaB represses E-cadherin expression and enhances epithelial to mesenchymal transition of mammary epithelial cells: potential involvement of ZEB-1 and ZEB-2. Oncogene.

[25] Heuberger J, Birchmeier W (2010). Interplay of cadherin-mediated cell adhesion and canonical Wnt signaling. Cold Spring Harb Perspect Biol.

[26] Saibil H (2013). Chaperone machines for protein folding, unfolding and disaggregation. Nat Rev Mol Cell Biol.

[27] Li XS, Xu Q, Fu XY, Luo WS (2014). Heat shock protein 60 overexpression is associated with the progression and prognosis in gastric cancer. PLoS One.

[28] Cappello F (2005). The expression of HSP60 and HSP10 in large bowel carcinomas with lymph node metastase. BMC Cancer.

[29] Zhou C (2018). Oncogenic HSP60 regulates mitochondrial oxidative phosphorylation to support Erk1/2 activation during pancreatic cancer cell growth. Cell Death Dis.

[30] Zhang J (2016). Hsp60 exerts a tumor suppressor function by inducing cell differentiation and inhibiting invasion in hepatocellular carcinoma. Oncotarget.

[31] Tsai YP (2009). Interaction between HSP60 and beta-catenin promotes metastasis. Carcinogenesis.

[32] Cheng MY (1989). Mitochondrial heat-shock protein hsp60 is essential for assembly of proteins imported into yeast mitochondria. Nature.

[33] Zhou J, Li XL, Chen ZR, Chng WJ (2017). Tumor-derived exosomes in colorectal cancer progression and their clinical applications. Oncotarget.

[34] Oloumi A, McPhee T, Dedhar S (2004). Regulation of E-cadherin expression and beta-catenin/Tcf transcriptional activity by the integrin-linked kinase. Biochim Biophys Acta.

[35] Chun JN (2010). Cytosolic Hsp60 is involved in the NF-kappaB-dependent survival of cancer cells via IKK regulation. PLoS One.

[36] Galvan JA (2015). Expression of E-cadherin repressors SNAIL, ZEB1 and ZEB2 by tumour and stromal cells influences tumour-budding phenotype and suggests heterogeneity of stromal cells in pancreatic cancer. Br J Cancer.

[37] Storci G (2010). TNFalpha up-regulates SLUG via the NF-kappaB/HIF1alpha axis, which imparts breast cancer cells with a stem cell-like phenotype. J Cell Physiol.

[38] Montserrat N (2011). Repression of E-cadherin by SNAIL, ZEB1, and TWIST in invasive ductal carcinomas of the breast: a cooperative effort?. Hum Pathol.

[39] Correction for Yu *et al*. Saliva protein biomarkers to detect oral squamous cell carcinoma in a high-risk population in Taiwan. *Proc Natl Acad Sci USA* **113**, E7139 (2016). 10.1073/pnas.1616695113 PMC5111674 27791196

[40] Solanas G (2008). E-cadherin controls beta-catenin and NF-kappaB transcriptional activity in mesenchymal gene expression. J Cell Sci.

[41] Kuphal S, Poser I, Jobin C, Hellerbrand C, Bosserhoff AK (2004). Loss of E-cadherin leads to upregulation of NFkappaB activity in malignant melanoma. Oncogene.

[42] Liu PF (2017). Vimentin is a potential prognostic factor for tongue squamous cell carcinoma among five epithelial-mesenchymal transition-related proteins. PLoS One.

[43] Chen W, Syldath U, Bellmann K, Burkart V, Kolb H (1999). Human 60-kDa heat-shock protein: a danger signal to the innate immune system. J Immunol.

[44] Grobe A (2014). Prognostic relevance of circulating tumor cells in blood and disseminated tumor cells in bone marrow of patients with squamous cell carcinoma of the oral cavity. Clin Cancer Res.

[45] Wong CS (2008). Identification of 5-fluorouracil response proteins in colorectal carcinoma cell line SW480 by two-dimensional electrophoresis and MALDI-TOF mass spectrometry. Oncol Rep.

[46] Fujii H (2009). Sphere-forming stem-like cell populations with drug resistance in human sarcoma cell lines. Int J Oncol.

[47] Fu TY (2016). Subsite-specific association of DEAD box RNA helicase DDX60 with the development and prognosis of oral squamous cell carcinoma. Oncotarget.

[48] Liu YP (2016). Suppressive function of low-dose deguelin on the invasion of oral cancer cells by downregulating tumor necrosis factor alpha-induced nuclear factor-kappa B signaling. Head Neck.

[49] Liu CW (2017). Histone Methyltransferase G9a Drives Chemotherapy Resistance by Regulating the Glutamate-Cysteine Ligase Catalytic Subunit in Head and Neck Squamous Cell Carcinoma. Mol Cancer Ther.

[50] Shu CW (2016). RelA-Mediated BECN1 Expression Is Required for Reactive Oxygen Species-Induced Autophagy in Oral Cancer Cells Exposed to Low-Power Laser Irradiation. PLoS One.

