# Author Correction: HSPD1 repressed E-cadherin expression to promote cell invasion and migration for poor prognosis in oral squamous cell carcinoma

**DOI:** 10.1038/s41598-020-58514-5

**Published:** 2020-01-30

**Authors:** Bor-Hwang Kang, Chih-Wen Shu, Jian-Kang Chao, Cheng-Hsin Lee, Ting-Ying Fu, Huei-Han Liou, Luo-Ping Ger, Pei-Feng Liu

**Affiliations:** 1 Department of Otorhinolaryngology-Head and Neck Surgery, Kaohsiung Veterans General Hospital, Kaohsiung, Taiwan; 2 Graduate Institute of Aerospace and Undersea Medicine, National Defense Medical Center, Taipei, Taiwan; 3 Department of Pharmacy, Tajen University, Pingtung, Taiwan; 4 School of Medicine for International Students, I-Shou University, Kaohsiung, Taiwan; 5 Institute of Biomedical Sciences, National Sun Yat-sen University, Kaohsiung, Taiwan; 6 Department of Psychiatry, Pingtung Branch, Kaohsiung Veterans General Hospital, Pingtung, Taiwan; 7 Department of Medical Education and Research, Kaohsiung Veterans General Hospital, Kaohsiung, Taiwan; 8 Department of Pathology and Laboratory Medicine, Kaohsiung Veterans General Hospital, Kaohsiung, Taiwan; 9 Department of Oral Hygiene, Shu-Zen Junior College of Medicine and Management, Kaohsiung, Taiwan

Correction to: *Scientific Reports* 10.1038/s41598-019-45489-1, published online 20 June 2019

This Article contains errors in Figure 1b, where the incorrect image is shown for TW1.5 cells transfected with scrambled siRNA at 0 h; Figure 1c, where the incorrect image is shown for TW1.5 cells transfected with scrambled siRNA; and in Figure 2e, where the incorrect image is shown for E-cadherin stable knockdown TW1.5 cells transfected with scrambled siRNA. The correct Figures 1 and 2 appear below.

**Figure 1:**
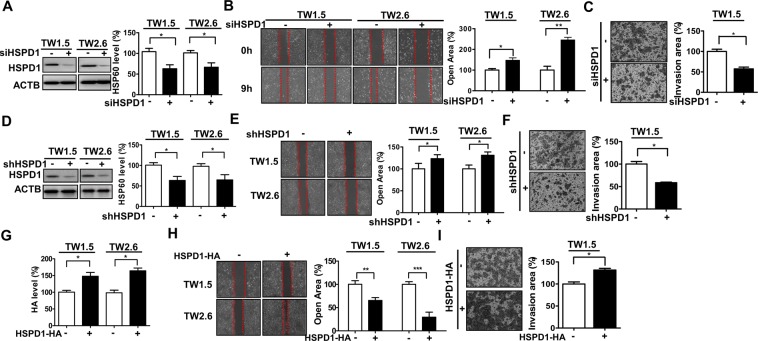
.

**Figure 2:**
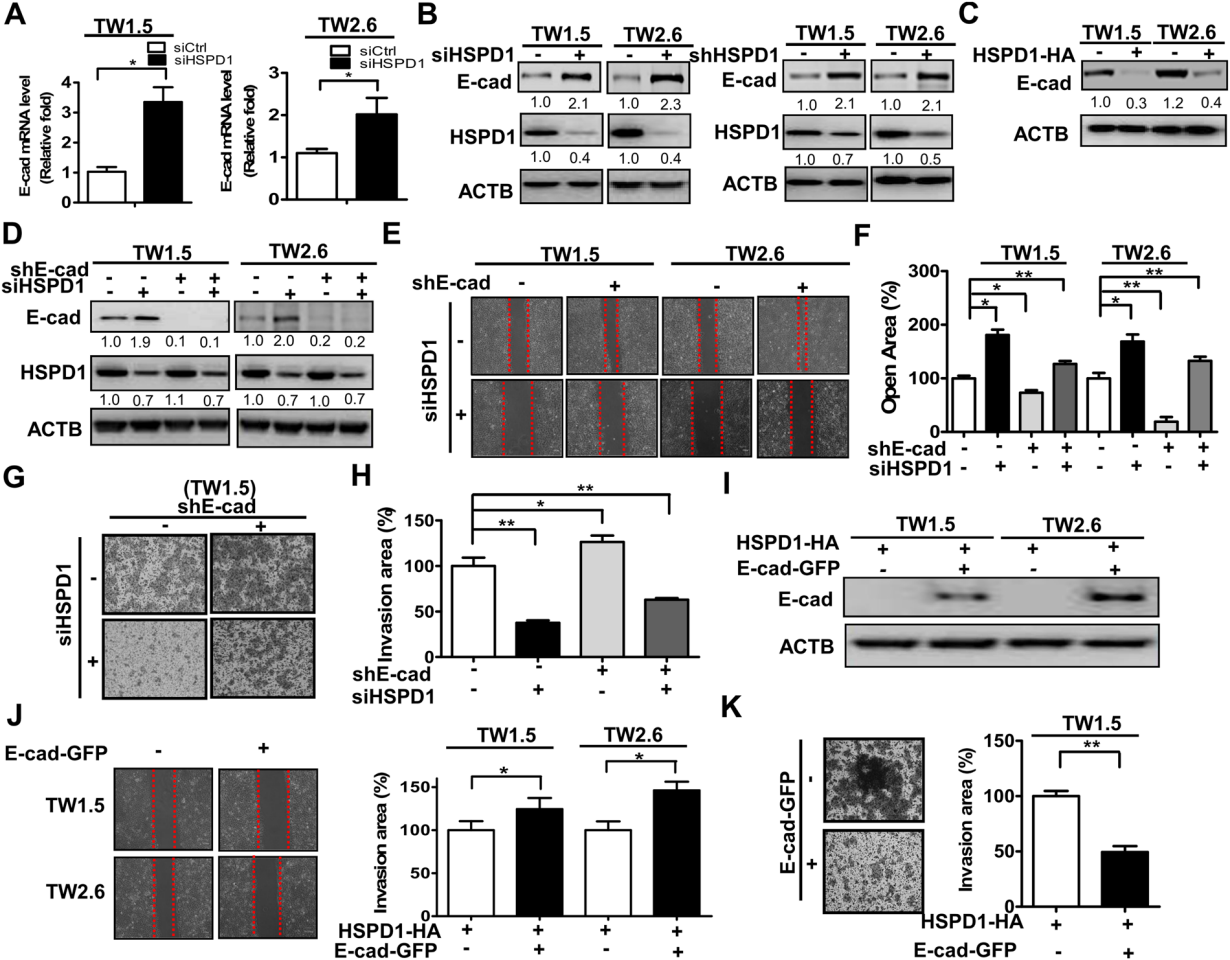
.

The conclusions of the Article are unaffected by these changes.

