# Supplementary material for: HSPD1 repressed E-cadherin expression to promote cell invasion and migration for poor prognosis in oral squamous cell carcinoma
Source: Sci Rep. 2019 Jun 20;9:8932. doi: 10.1038/s41598-019-45489-1 (PMC6586902; doi:10.1038/s41598-019-45489-1)

Supplementary Information

HSPD1 repressed E-cadherin expression to promote cell invasion and migration for poor prognosis in oral squamous cell carcinoma

Bor-Hwang Kang, Chih-Wen Shu, Jian-Kang Chao, Cheng-Hsin Lee, Ting-Ying Fu, Huei-Han Liou , Luo-Ping Ger, Pei-Feng Liu

Contents:

Supplementary Figures (S1, S2 and S3)

Supplementary Table (S1, S2, S3, S4, S5 and S6)

Full length blots for each Figure

Supplementary Fig. S1

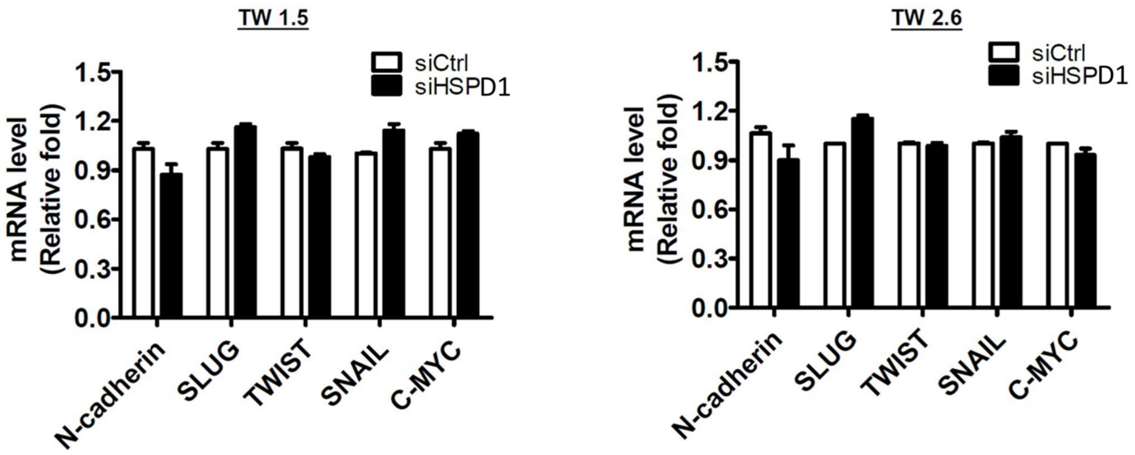

Supplementary Fig. S1. Transcriptional changes of EMT-related genes in HSPD1-knockdown TW1.5 and TW2.6 cells by RT-PCR.

Supplementary Fig. S2

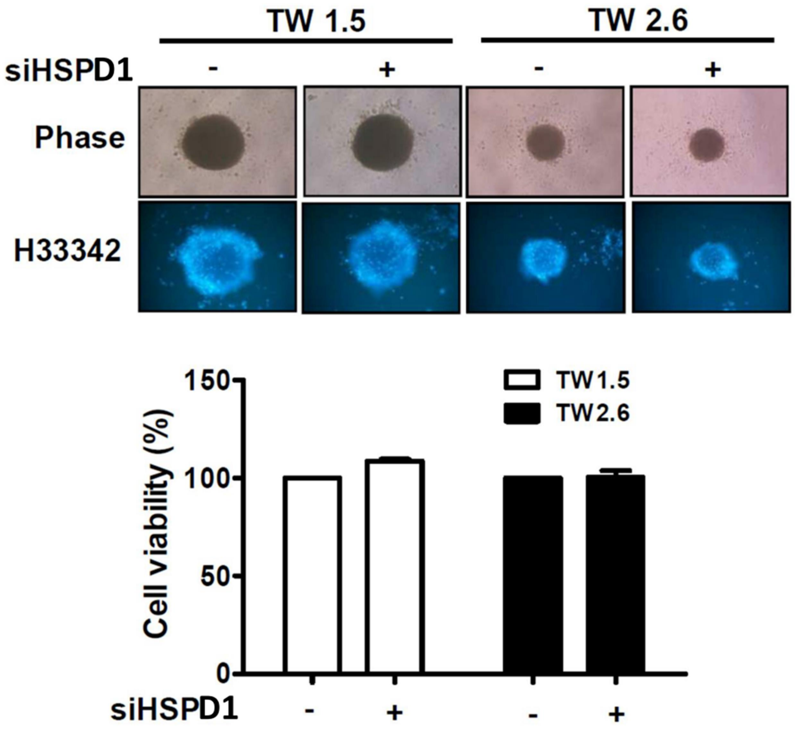

Supplementary Fig. S2. Tumor viability of spheroids with HSPD1 knockdown via siRNA for 48 h. The spheroid cells were lysed to measure ATP level for cell viability.

Supplementary Fig. S3

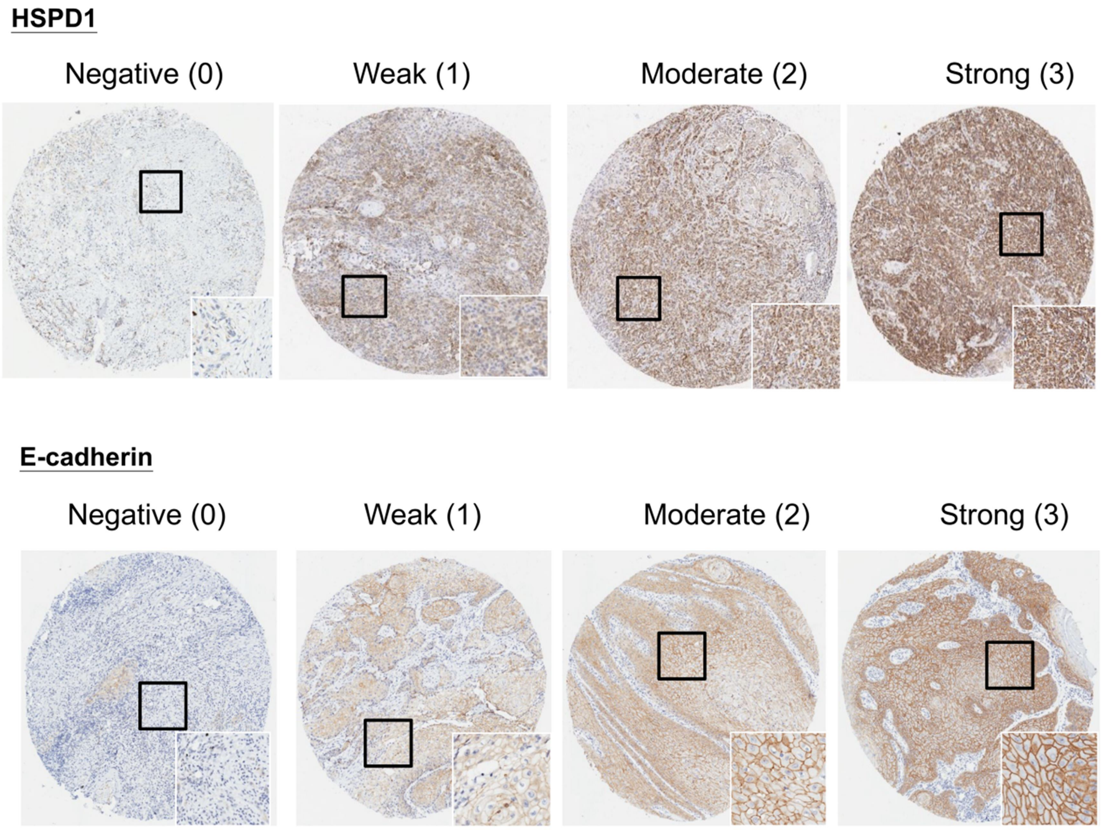

Supplementary Fig. S3. Immunoreactivity of HSPD1 and E-cadherin in BMSCC. The representative immunohistochemical staining intensity of proteins as negative (0), weak (1), moderate (2), and strong (3).

Supplementary Table S1. The comparison of HSPD1 and E-cadherin protein levels between corresponding tumor adjacent normal tissues and tumor tissues in BMSCC patients

Variables	No.	Tumor adjacent normal		Tumor		Z	p value*
		Mean±SD	Median	Mean±SD	Median		
**HSPD1**	128	2.95±1.34	3.00	4.38±1.27	4.00	7.462	**<0.001**
**E-cadherin**	128	5.34±1.55	5.50	3.52±1.31	4.00	7.952	**<0.001**

Abbreviations: BMSCC, Buccal mucosa squamous cell carcinoma; SD, Standard deviation.
***p values were estimated by Wilcoxon signed-rank test.**

Supplementary Table S2. The impact of co-expression level of HSPD1 and E-cadherin in overall survival of BMSCC patients

**Variable**	No. (%)	CHR (95% CI)	p value*	AHR (95% CI)	p value†
**Overall survival**					
**HSPD1 (L) E-cad(H)**	66 (35.5)	1.00		1.00	
**HSPD1 (L) E-cad(L)**	28 (15.1)	0.87 (0.50-1.53)	0.635	1.06 (0.57-1.98)	0.862
**HSPD1 (H) E-cad(L)**	57 (30.6)	1.94 (1.30-2.88)	**0.001**	1.88 (1.18-2.98)	**0.008**
**HSPD1 (H) E-cad(H)**	35 (18.8)	0.67 (0.40-1.12)	0.125	0.86 (0.48-1.54)	0.604

Abbreviations: BMSCC, buccal mucosal squamous cell carcinoma; CHR, crude hazard ratio; CI, confidence interval; AHR, adjusted hazard ratio.

***p values were estimated by Cox's regression.**

†p values were adjusted for cell differentiation (moderate+poor vs. well) and AJCC pathological stage (stage III+ IV vs stage I+II) by multivariate Cox's regression.

Supplementary Table S3. The impact of co-expression level of HSPD1 and E-cadherin in survival of oral cancer patients from TCGA database					
**Variables**	No. (%)	CHR (95% CI)	p value*	AHR (95% CI)	p value†
**Overall survival**					
**HSPD1 (L) E-cad (H)**	79 (26.3)	1.00		1.00	
**HSPD1 (H) E-cad (L)**	79 (26.3)	**1.66 (1.14-2.43)**	**0.008**	**1.86 (1.15-3.01)**	**0.012**
**HSPD1 (H) E-cad (H)**	71 (23.7)	1.14 (0.76-1.71)	0.516	1.42 (0.86-2.36)	0.175
**HSPD1 (L) E-cad (L)**	71 (23.7)	0.70 (0.45-1.08)	0.109	0.96 (0.56-1.64)	0.873
**Disease-free survival**					
**HSPD1 (L) E-cad (H)**	55 (25.7)	1.00		1.00	
**HSPD1 (H) E-cad (L)**	55 (25.7)	1.63 (0.91-2.94)	0.101	2.12 (0.94-4.75)	0.069
**HSPD1 (H) E-cad (H)**	52 (24.3)	0.58 (0.27-1.23)	0.157	0.96 (0.37-2.50)	0.940
**HSPD1 (L) E-cad (L)**	52 (24.3)	1.46(0.81-2.64)	0.213	1.96 (0.87-4.43)	0.107

Abbreviations: AHR, Adjusted hazard ratio; CHR, Crude hazard ratio; CI, Confidence interval; E-cad, E-cadherin; H, High expression; L, Low expression.
***p values were estimated by Cox's regression.**
†p values were estimated by multivariate Cox's regression.

**Supplementary Table S4. The impact of co-expression level of HSPD1 and RelA in survival of oral cancer patients from TCGA database**					
**Variable**	No. (%)	CHR (95% CI)	p value*	AHR (95% CI)	**p value†**
**Overall survival**					
**HSPD1 (L) RelA (L)**	58 (19.3)	1.00		1.00	
**HSPD1 (H) RelA (H)**	58 (19.3)	1.50 (1.00-2.26)	0.051	**1.98 (1.11-3.55)**	**0.021**
**HSPD1 (H) RelA (L)**	92 (30.7)	1.31 (0.90-1.90)	0.156	1.74 (1.00-3.02)	0.051
**HSPD1 (L) RelA (H)**	92 (30.7)	0.74 (0.50-1.10)	0.141	1.16 (0.66-2.06)	0.608
**Disease-free survival**					
**HSPD1 (L) RelA (L)**	44 (20.6)	1.00		1.00	
**HSPD1 (H) RelA (H)**	44 (20.6)	0.67 (0.31-1.42)	0.296	0.71 (0.28-1.76)	0.454
**HSPD1 (H) RelA (L)**	63 (29.4)	1.43 (0.79-2.56)	0.236	1.25 (0.59-2.68)	0.559
**HSPD1 (L) RelA (H)**	63 (29.4)	0.92(0.50-1.71)	0.790	0.92 (0.42-2.03)	0.841

Abbreviations: AHR, Adjusted hazard ratio; CHR, Crude hazard ratio; CI, Confidence interval; H, High expression; L, Low expression.
***p values were estimated by Cox's regression.**
†p values were estimated by multivariate Cox's regression.

Supplementary Table S5. The expression correlation of HSPD1 and EMT-related markers in BMSCC patients

	EMT markers				
	Vim	N-cad	E-cad	Snail	Twist
**HSPD1**	r = -0.033	r = 0.018	r = -0.327	r = 0.018	r = 0.049
	p = 0.660	p = 0.814	**p < 0.001**	p = 0.809	p = 0.515

Abbreviations: BMSCC, Buccal mucosal squamous cell carcinoma; EMT, epithelial–mesenchymal transition;Vim, Vimentin; E-cad, E-cadherin; N-cad, N-cadherin.
The correlation coefficient and p-value were estimated by Spearman rank correlation coefficient.

Supplementary Table S6. Correlation between HSPD1 protein level and three risk factors in BMSCC patients

Risk factors of BMSCC	HSPD1 (Low level)	HSPD1 (High level)	p-value
	Number (%)	Number (%)	
**BQ chewing (n=116)**			
**No**	12 (13.0)	25 (27.2)	**0.009**
**Yes**	33 (35.9)	22 (23.9)	
**Smoking (n=116)**			
**No**	12 (10.3)	16 (13.8)	0.976
**Yes**	38 (32.8)	50 (43.1)	
**Drinking (n=115)**			
**No**	20 (17.4)	29 (25.2)	0.738
**Yes**	29 (25.2)	37 (32.2)	

Abbreviations: BMSCC, Buccal mucosa squamous cell carcinoma; BQ, Betal quid.
*p values were estimated by chi-square test.

Full length blots for each Figure

Fig. 1A

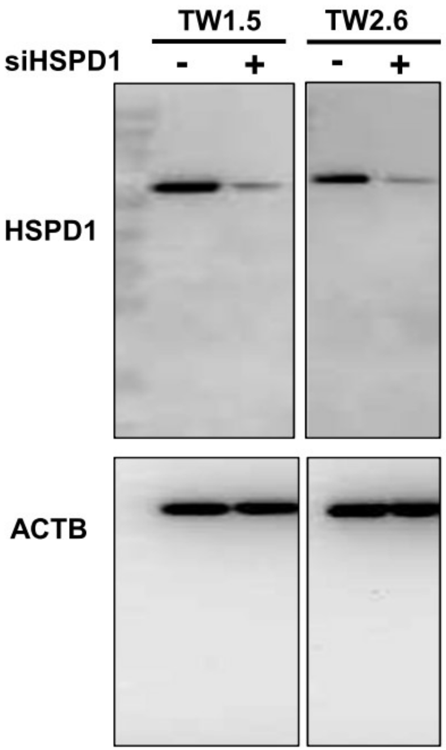

Fig. 1D

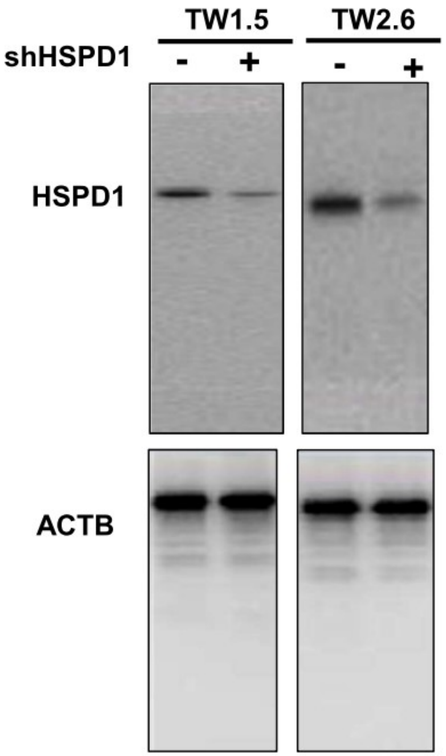

Fig. 1G

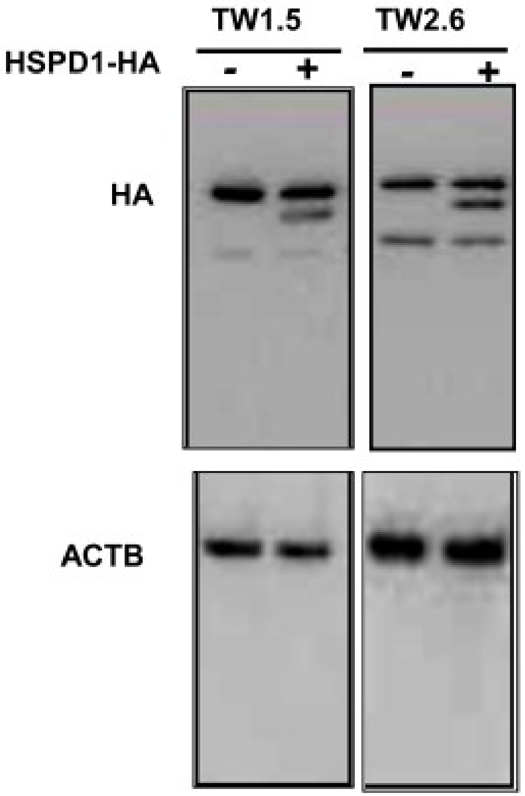

Full length blots for each Figure

Fig. 2B

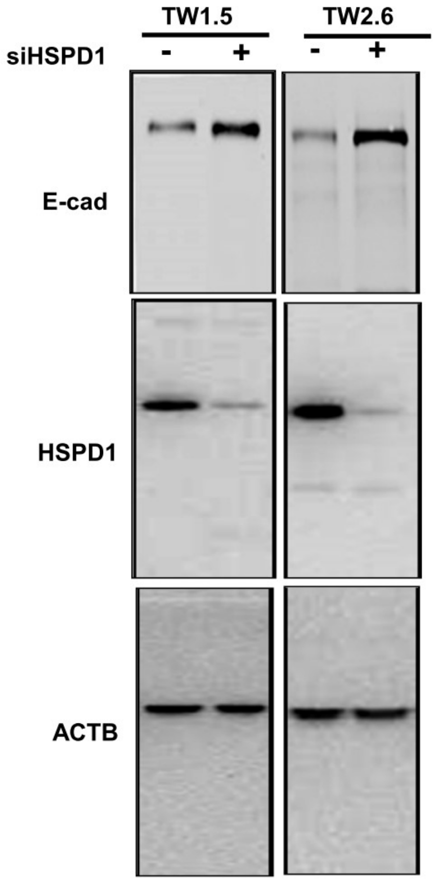

Fig. 2B

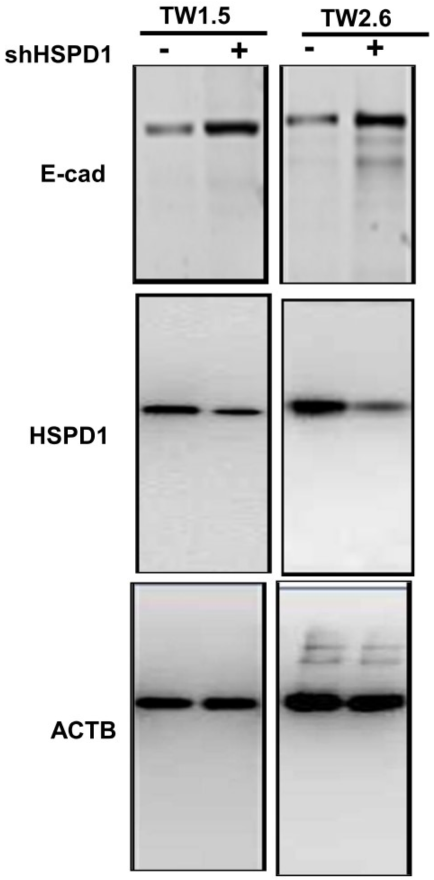

Fig. 2C

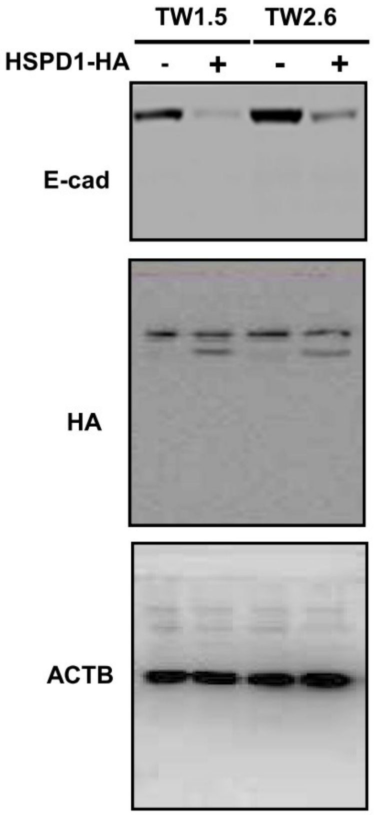

Full length blots for each Figure

Fig. 2D

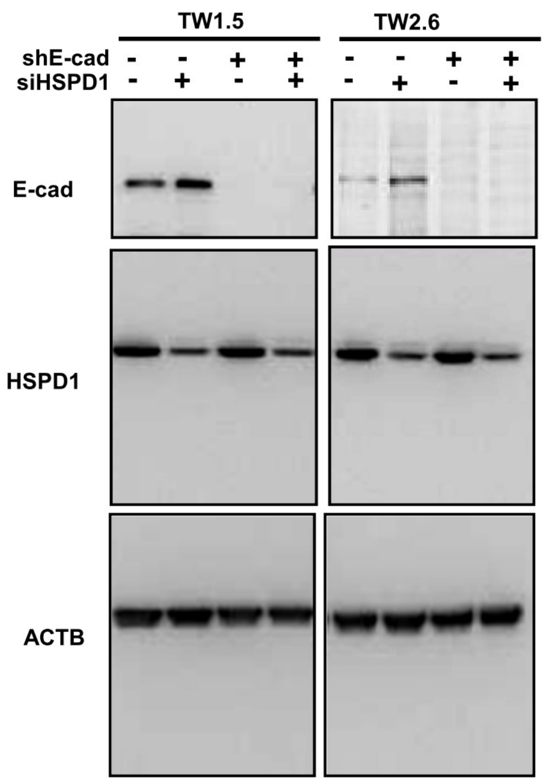

Fig. 2I

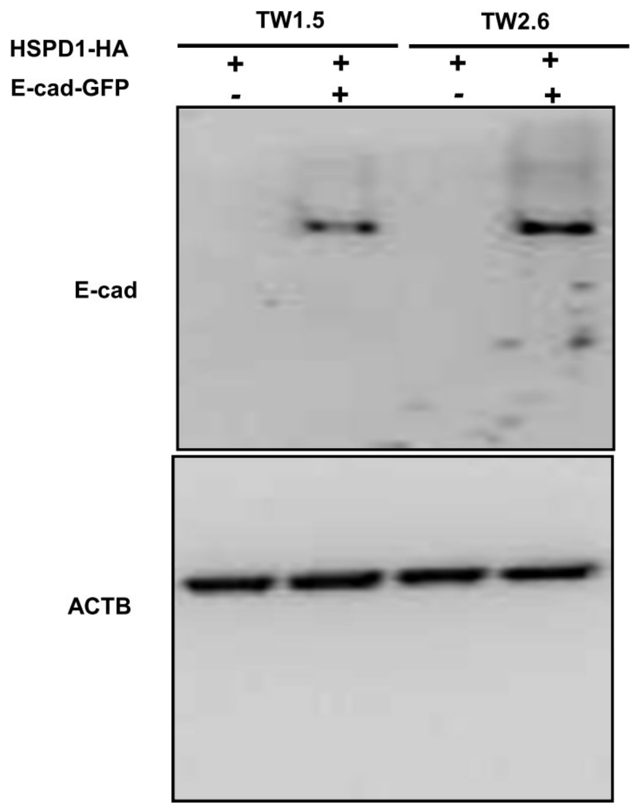

Full length blots for each Figure

Fig. 3A

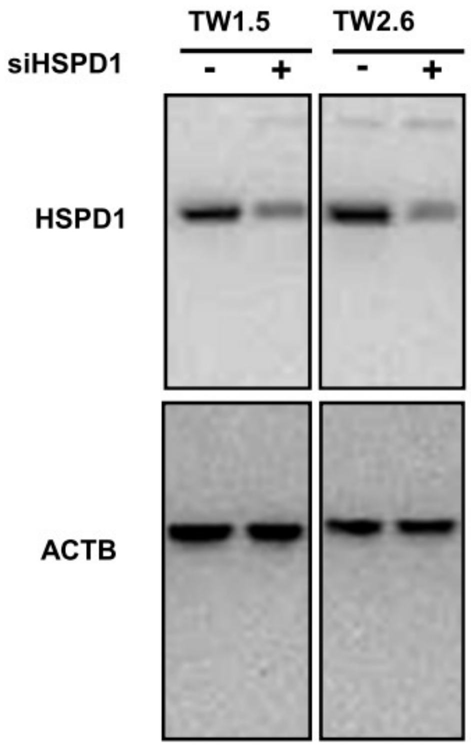

Fig. 3C

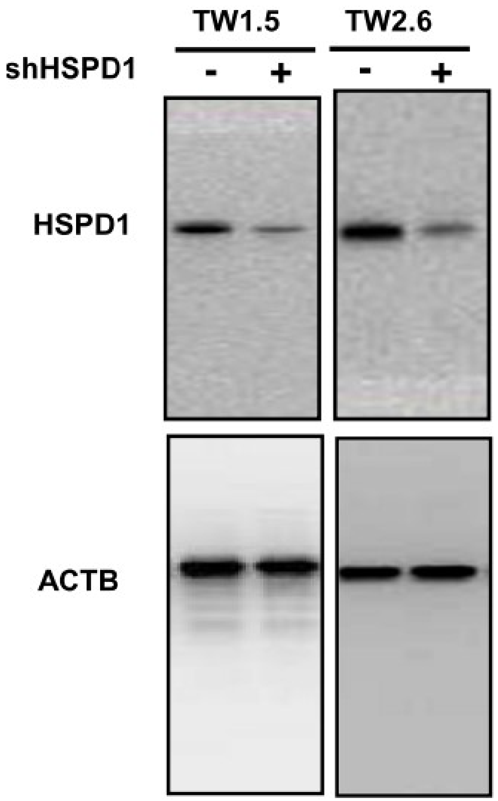

Fig. 3E

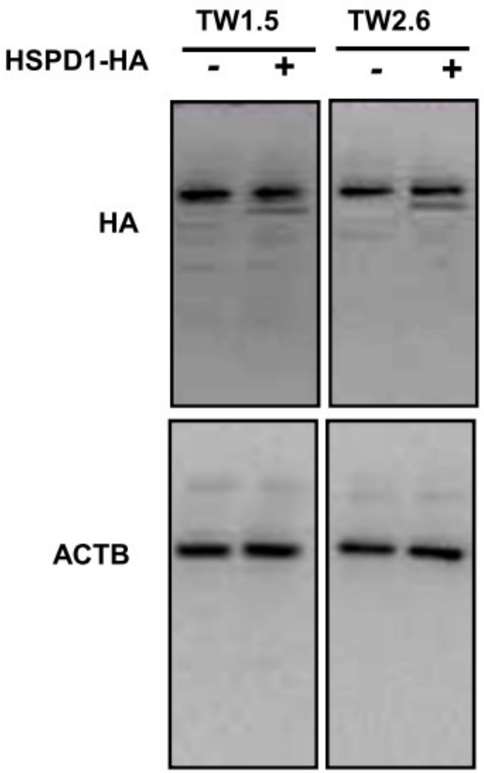

Full length blots for each Figure

Fig. 4A

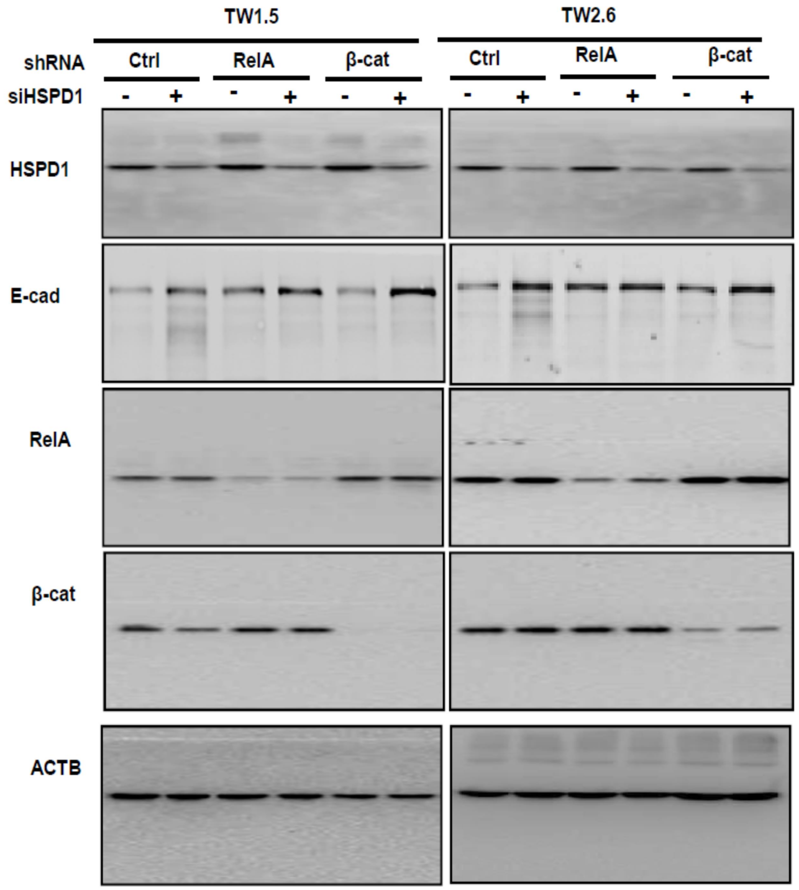

Fig. 4F

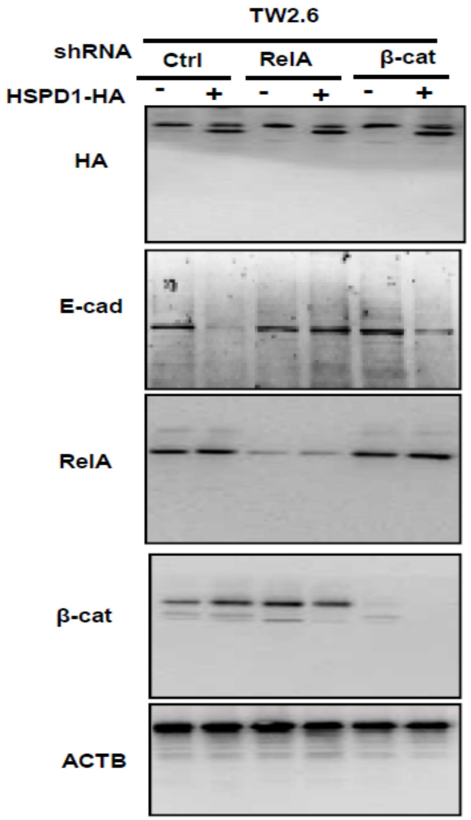

Full length blots for each Figure

Fig. 4I

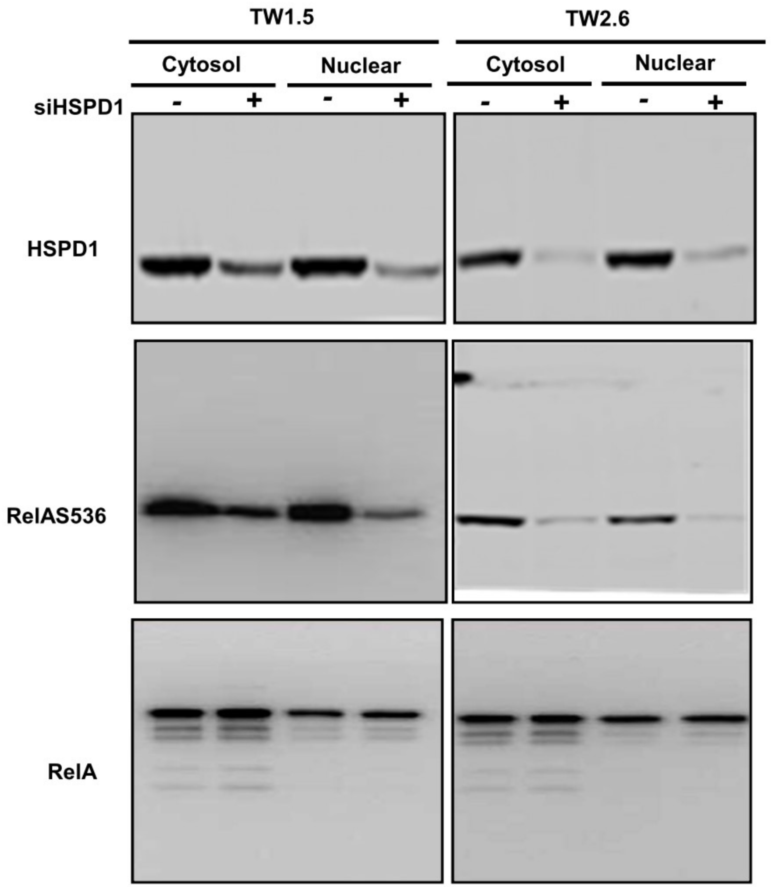

Fig. 4I

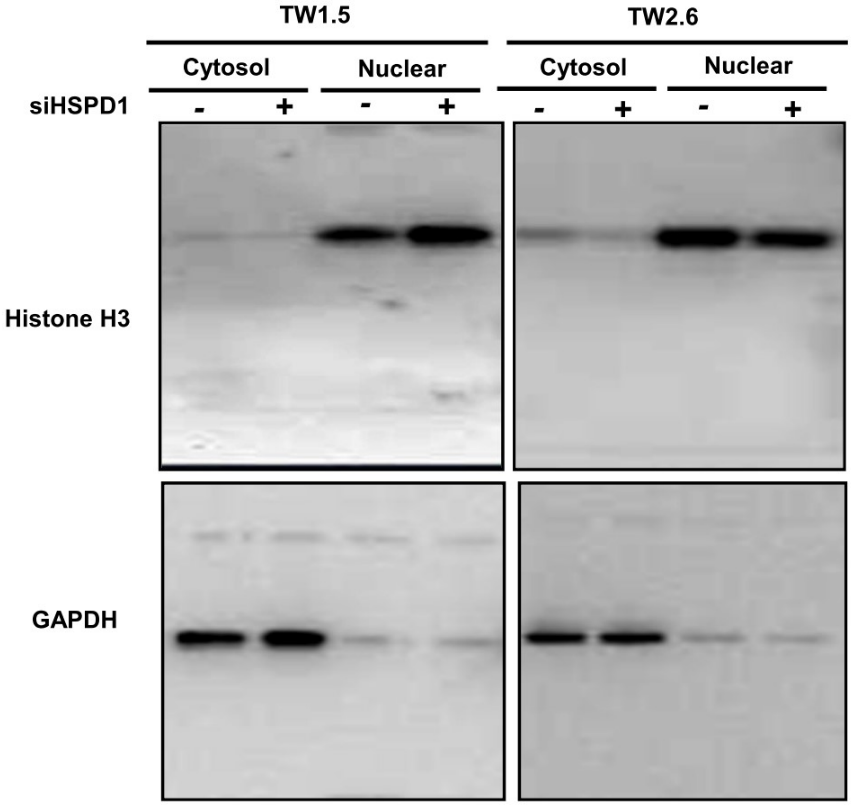

Supplement: Supplementary file 1 — Supplementary Information [file 41598_2019_45489_MOESM1_ESM.pdf]
